# Development and Applications of a Magnetism‐Aware General‐Purpose Atomic Cluster Expansion Potential for the Fe‐O‐H Ternary System

**DOI:** 10.1002/advs.76709

**Published:** 2026-07-27

**Authors:** Baptiste Bienvenu, Mira Todorova, Jörg Neugebauer, Dierk Raabe, Matous Mrovec, Ralf Drautz

**Affiliations:** ^1^ Max Planck Institute for Sustainable Materials Düsseldorf Germany; ^2^ Interdisciplinary Centre for Advanced Materials Simulation Ruhr‐Universität Bochum Bochum Germany

**Keywords:** cluster expansion, hydrogen embrittlement, hydrogen, interatomic potential, magnetism, metallurgy

## Abstract

We develop and benchmark a general‐purpose machine‐learned interatomic potential for the Fe‐O‐H ternary system, based on the Atomic Cluster Expansion. Following our approach developed for the Fe‐O system, magnetism is explicitly treated by the model in an Ising‐like manner. This allows efficient incorporation of magnetic degrees of freedom, making the potential applicable to large‐scale atomistic calculations. We demonstrate the capability of the model to accurately describe a wide range of properties and capture basic mechanisms underlying hydrogen‐based reduction of iron oxides, interaction of water with iron and hydrogen embrittlement of metallic iron.

## Graphical abstract

Hydrogen‐driven iron oxide reduction, water‐iron corrosion, and hydrogen embrittlement of metallic iron share the same Fe‐O‐H chemistry. A general‐purpose Atomic Cluster Expansion potential covers this chemistry across the full concentration range with explicit Ising‐like treatment of magnetism. The model addresses all three problems at the atomistic scale, enabling simulations far beyond the reach of first‐principles methods.

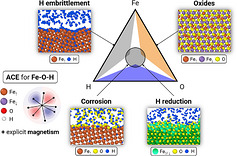

## 1 Introduction

The iron‐oxygen‐hydrogen (Fe‐O‐H) ternary system hosts a wide variety of technologically relevant applications, ranging from corrosion [1, 2], extraction of metallic iron from hydrogen‐based oxide reduction [3], hydrogen embrittlement [4, 5], to catalytic water splitting [6, 7]. Atomic scale rationalization and understanding of the mechanisms underlying these phenomena is of great interest, thus motivating atomistic simulations.

Conducting such simulations often involves time and length scales not easily accessible to versatile and accurate ab initio calculations. This motivates the development of an efficient and transferable interatomic potential that covers the entire concentration range of both oxygen (O) and hydrogen (H). To our knowledge, such a potential is currently unavailable in the literature. Performing atomistic simulations at the very scale at which mechanisms underlying the aforementioned processes occur, then allows to design pathways to stepwise analyze, understand and optimize these processes, down to scales not easily accessible to experiments.

Magnetism, a fundamental attribute of elemental iron, its oxides, and its magnetic alloys, is often rigorously treated within the framework of density functional theory (DFT); yet, it remains frequently overlooked in the construction of interatomic potentials. Developments have been made in embedding magnetic degrees of freedom within interatomic potentials [8, 9], including recent machine‐learned potentials, with different degrees of approximation [10, 11, 12]. Such models have been successfully applied to a variety of magnetic materials, ranging from elemental Fe with a non‐collinear magnetic model [13], to our previous work on the Fe‐O binary system [14], with an Ising‐like model to treat magnetic degrees of freedom.

Here, we introduce a magnetism‐aware Atomic Cluster Expansion (ACE) [15] for the ternary Fe‐O‐H system, validated against DFT calculations across a wide range of material properties. We then demonstrate the model's ability to capture the fundamental mechanisms of hydrogen‐based iron oxide reduction, hydrogen embrittlement, and water‐iron surface interactions.

## 2 Computational Details

### 2.1 Ab Initio Calculations

All ab initio calculations performed in this work, both for fitting the ACE potential and validation of various properties, were carried out within the framework of DFT as implemented in the vasp code [16], with the same parameters as in our previous work on the Fe‐O system [14]. Projector‐augmented wave pseudo‐potentials are used to model Fe, O and H atoms, including 10, 6 and 1 valence electrons, respectively. Exchange and correlation (xc) are approximated by the generalized‐gradient approximation (GGA), in the method developed by Perdew et al. (GGA‐PBE) [17]. A plane‐wave basis set with an energy cutoff of 500 eV with a Γ‐centered k‐point mesh of density $0.02\;2\pi \;{Å}^{-1}$ were used. Magnetism is treated in the collinear approximation, within spin‐polarized DFT.

As discussed in our previous work focusing on the development of an interatomic potential for the binary Fe‐O system [14], DFT GGA functionals yield a rather poor description of the electronic properties of iron oxides. However, other DFT variants (also including meta‐GGA functionals) cannot properly describe pure Fe, iron hydrides, as well as Fe with dilute concentrations of O or H, which are all relevant for the development of a potential covering such large regions of the compositional range. Our choice of the GGA‐PBE functional therefore enables to achieve a consistent description of Fe atoms across the whole range of O and H concentrations.

### 2.2 ACE Potential Training and Fitting

The training DFT data, which contains a total of 122000 structures (see more details about the content in Supporting Information, Note S9), encompass the whole range of O and H concentrations from pure Fe to pure O and H, including combinations of O and H only. During the generation of training data, more emphasis was put on pure iron, iron oxides ${\mathrm{Fe}}_{x}O_{y}$, iron hydroxides ${\text{Fe}}_{x}{\left(\text{OH}\right)}_{y}$, and H‐containing structures (of various concentrations), as well as stoichiometric iron hydrides. Most of the training set consists of random structures spanning all space‐group symmetries and ranges of stoichiometry, generated using both the pyxtal [18] and buildcell [19] codes, following the ASSYST method proposed in Refs. [20, 21]. This methodology, previously employed in the development of our binary Fe‐O ACE potential [14], facilitates comprehensive sampling of the configurational space. Consequently, the resulting potential exhibits high robustness across a diverse array of compositions, transformations and applications, including those involving extreme environments. To complement these structural and compositional variations, we incorporated diverse collinear magnetic arrangements for structures containing Fe, thereby accounting for magnetic degrees of freedom.

**FIGURE 1:**
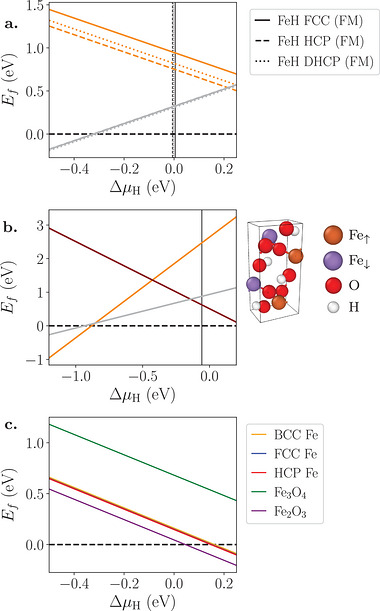
Vacancy formation energies $E_{f}$ in: (a) Fe hydrides FeH and (b) FeO(OH). For FeH (a), $\mu_{H}$ is referenced to molecular $H_{2}$, while for FeO(OH) (b), $\mu_{O}$ is referenced to H through the stability of $H_{2}O$ (i.e. $E_{H_{2}O}=2\mu_{H}+\mu_{O}$), and $\mu_{H}$ is also referenced to molecular $H_{2}$. (c) Formation energies $E_{f}\left(H_{\text{int.}}\right)$ of an interstitial H atom in the three Fe polymorphs (BCC, FCC and HCP) and the two ${\mathrm{Fe}}_{3}O_{4}$ and ${\mathrm{Fe}}_{2}O_{3}$ iron oxides, where $\mu_{H}$ is referenced to molecular $H_{2}$. Vertical black lines in (a,b) indicate the stability range of the bulk material.

We base our ternary Fe‐O‐H model on ACE, as in our previous work on the Fe‐O binary system, which offers a very good balance between accuracy and computational efficiency (see for instance Figure 1 in the work of Lysogorskiy et al. [22]), but also motivated by its clear physical justification. The fitted ACE potential explicitly includes magnetic degrees of freedom in an Ising‐like fashion, as proposed in our previous work on the Fe‐O system [14], and also used recently for stainless ${\mathrm{Fe}}_{7}{\mathrm{Cr}}_{2}\mathrm{Ni}$ austenitic steels [23]. Such a definition allows efficient model parameterization, reducing both the amount of training data required and the model complexity (e.g., compared with explicit non‐collinear formalisms for magnetic degrees of freedom [9, 13]), and is thus suitable for large‐scale atomistic simulations. As discussed in our previous work on the Fe‐O potential [14], all interactions are enforced to account for the spin‐inversion symmetry.

The ternary model is based on the existing binary Fe‐O ACE potential, supplemented with additional interaction parameters for H‐containing interactions. A cutoff of 7 Å is used for binary interactions between Fe and O (i.e. Fe‐Fe, Fe‐O and O‐O), and of 5 Å for all interactions involving H. The final model contains 4955 functions and 12046 parameters. The overall accuracy of the resulting ACE model, in terms of the mean average error with respect to the DFT reference, is 17.3 meV/atom in energy and 68.4 meV/Å in forces. Even though the fitting of the potential started using previously optimized parameters for binary Fe‐O interactions, these parameters slightly changed during optimization. Overall, properties predicted over the Fe‐O binary sub‐system by this new potential are in very good agreement with those reported for the Fe‐O potential in our previous work [14] (see validation in Supporting Information).

We also note that interactions involving non‐magnetic Fe atoms, which were included in the binary Fe‐O potential [14], are not supported by the ternary Fe‐O‐H ACE potential. This choice helps reduce the complexity of the model and its training, and is also motivated by the lesser importance of these interactions with respect to the target applications of the ternary potential.

## 3 Results and Discussion

### 3.1 Validation of the Potential

In this section, we validate the proposed ACE potential against the fundamental material properties of representative phases within the Fe‐O‐H system. Details about simulation setup (geometry, parameters, number of atoms) and methods can be found in Supporting Information.

#### 3.1.1 Fundamental Properties

We first focus on fundamental bulk properties of the main physically relevant materials that were not considered in our preceding work on the binary Fe‐O system, i.e. iron hydrides and hydroxides, presented in Table 1.

**TABLE 1:** Bulk properties of iron hydrides FeH (FCC, HCP and DHCP phases), and iron hydroxide FeO(OH) (goethite): lattice parameters a, b, c (in Å), bulk modulus $B_{0}$ (in GPa), and formation enthalpy $\Delta H_{f}$ (in eV/atom); for the $H_{2}$ molecule, its equilibrium bond distance $d_{\text{eq.}}$ (in Å) and binding energy $E_{b}$ (in eV); density of liquid $H_{2}O$ ρ (in g/${\mathrm{cm}}^{3}$) at 300 K, compared to a recent ACE potential for water [24]. Predictions of the ACE potential are compared to DFT and experimental references, if computed in this work or available from literature. FM: ferromagnetic; AF: antiferromagnetic.

	ACE	DFT	Expt.
**FeH FCC (FM)**			
a	3.75	3.77	/
$B_{0}$	217.0	/	/
$\Delta H_{f}$	+0.002	+0.010	/
**FeH HCP (FM order)**			
(a, c)	(2.64, 8.74)	(2.65, 8.67)	/
$B_{0}$	166.2	/	/
$\Delta H_{f}$	−0.004	−0.002	/
**FeH DHCP (FM order)**			
(a, c)	(2.64, 8.72)	(2.65, 8.68)	/
$B_{0}$	187.5	/	/
$\Delta H_{f}$	−0.001	−0.001	/
**FeO(OH) (AF order)**			
(a, b, c)	(3.05, 4.72, 9.84)	(3.02, 4.65, 10.02)	
$B_{0}$	63.2	/	/
$\Delta H_{f}$	−1.174	−1.168	/
$H_{2}$ **molecule**			
$d_{\text{eq.}}$	0.75	0.75	0.74
$E_{b}$	4.52	4.52	4.52
**Liquid** $H_{2}O$			
ρ	0.89 (*0.94* [24])	/	1.00

In light of the minor refinements to the Fe‐O parameters within the ternary potential, we have rigorously verified that the fundamental properties of iron oxides and iron polymorphs remain consistent, both with the DFT benchmarks and with predictions of our existing binary ACE potential [14]. These comparative data are detailed in the Supporting Information.

All polymorphs of Fe hydrides FeH (i.e. FCC, HCP and DHCP) are predicted by both the ACE potential and DFT calculations to have a ferromagnetic (FM) ground state, while goethite FeO(OH) is predicted as an antiferromagnetic (AF) phase. We also report a very good agreement with the DFT reference in terms of the basic properties presented in Table 1 (lattice parameters, bulk moduli, formation enthalpies). The relative stability of the three stoichiometric iron hydrides FeH is well captured by the model, as well as the equilibrium and elastic properties of goethite, FeO(OH). Additional properties, such as phonon spectra, are provided in the Supporting Information.

#### 3.1.2 Properties of Simple Defects

Accurate description of point defect properties is essential for simulations of diffusion processes and stoichiometry deviations frequently observed in iron oxides, hydrides, and hydroxides. Both phenomena are intrinsically governed by the mobility and concentration of point defects that are sensitive to the surrounding chemical environment [25].

We investigated properties of simple defects, such as vacancies and interstitials, in various bulk phases within the Fe‐O‐H system, i.e. Fe polymorphs (BCC, FCC and HCP), Fe oxides (${\mathrm{Fe}}_{3}O_{4}$ and ${\mathrm{Fe}}_{2}O_{3}$), Fe hydrides (FeH), and the stable iron hydroxide goethite FeO(OH). Predicted formation energies of these defects as a function of the H chemical potential $\Delta \mu_{H}$ are shown in Figure 1.

We stress that we only focus on charge‐neutral defects since neither our ACE model nor the underlying DFT data used for its training capture effects related to charged defect configurations. Therefore, the computed formation energies are expected to be representative of the high temperature range only, where charge effects are smeared out and the structural aspects dominate. At low temperatures, the relative stability of point defects can strongly depend on their charge states, especially in Fe oxides [26, 27]. Nevertheless, Banerjee et al. [27] also reported only a marginal influence of the charge state on the vacancy migration barriers in ${\mathrm{Fe}}_{2}O_{3}$, stressing that the neutral approximation may be valid in some cases.

As shown in Figure 1a, vacancy formation energies have similar magnitudes for both the Fe and H sub‐lattices in the three polymorphs of FeH, with $V_{\text{Fe}}$ being the lowest in energy in the HCP phase. The Fe hydrides, like Fe oxides, often exhibit stoichiometric deviations from the nominal FeH composition depending on the ambient conditions (temperature and pressure) [28, 29]. A similar picture applies to goethite FeO(OH) (see Figure 1b).

We also computed the solution energy of an interstitial H atom ($H_{\text{int.}}$) in the three polymorphs of pure Fe and the two Fe oxides ${\mathrm{Fe}}_{3}O_{4}$ and ${\mathrm{Fe}}_{2}O_{3}$, presented in Figure 1c as a function of $\Delta \mu_{H}$. In pure Fe, the solution energy for a H atom $E_{f}\left(H_{\text{int.}}\right)$ is the highest in the BCC phase, followed by the FCC and HCP phases, in which $E_{f}\left(H_{\text{int.}}\right)$ is almost the same. For the two Fe oxides, $E_{f}\left(H_{\text{int.}}\right)$ is much higher in ${\mathrm{Fe}}_{3}O_{4}$ than in ${\mathrm{Fe}}_{2}O_{3}$, by approximately 0.6 eV. A comparison of all considered defect energies predicted by the ACE potential with those obtained by the DFT calculations is provided in Table 2. Overall, there is a good agreement for most values with larger deviations observed only for goethite.

**TABLE 2:** Comparison between predictions of the ACE potential and DFT calculations (italic) for point defect formation energies $E_{f}$ (in eV) in various prototype materials. H is referenced to molecular $H_{2}$, and O to molecular $O_{2}$, except in FeO(OH), where equilibrium of $H_{2}O$ is used to link H and O chemical potentials, as in Figure 1.

Material	$E_{f}\left(V_{\text{Fe}}\right)$	$E_{f}\left(V_{O}\right)$	$E_{f}\left(V_{H}\right)$
FeH FCC	0.95 (*0.75*)	/	0.32 (*0.12*)
FeH HCP	0.75 (*1.10*)	/	0.32 (*0.24*)
FeH DHCP	0.82 (*0.99*)	/	0.31 (*0.27*)
FeO(OH)	2.64 (*3.74*)	0.51 (*0.53*)	0.93 (*1.54*)

Material	$E_{f}\left(H_{\text{int.}}\right)$		
Fe BCC	0.16 (*0.20*)		
Fe FCC	0.15 (*0.15*)		
Fe HCP	0.15 (*0.19*)		
${\mathrm{Fe}}_{3}O_{4}$	0.68 (*0.67*)		
${\mathrm{Fe}}_{2}O_{3}$	0.05 (*0.19*)		

For the same reason as for the basic properties in the previous section, since the binary Fe‐O interactions have changed with respect to our previous work [14], we recomputed point defect properties for the three Fe phases and Fe oxides. These results (vacancy formation energies and diffusion barriers) are presented in the Supporting Information.

### 3.2 Hydrogen Trapping and Diffusion

Interstitial H atoms can readily diffuse in the bulk Fe matrix, especially in BCC Fe where the H migration barrier is very low [30]. In real materials, H atoms get often trapped at various defects [31, 32, 33], altering their intrinsic properties and resulting in undesired macroscopic phenomena such as hydrogen embrittlement [34]. It is thus important that the ACE potential correctly describes the interactions of H with crystal defects.

#### 3.2.1 Hydrogen Trapping at Vacancies

**FIGURE 2:**
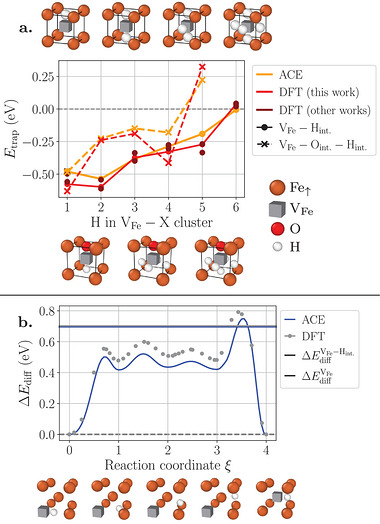
(a) Incremental trapping energy $E_{\text{trap}}$ of interstitial H atoms ($H_{\text{int.}}$) at a single Fe vacancy ($V_{\text{Fe}}-H_{\text{int.}}$) and a $V_{\text{Fe}}-O_{\text{int.}}-H_{\text{int.}}$ complex in BCC Fe, predicted by the ACE potential and DFT calculations, from this work, and previous works [35, 36]. Different configurations are represented in the top panel. (b) Energy profile for the coupled diffusion of a $V_{\text{Fe}}-H_{\text{int.}}$ complex in BCC Fe. Predictions of the ACE potential are compared to DFT data from Ref. [35]. Zero point energy corrections were not accounted for.

We first focus on trapping of interstitial H atoms ($H_{\text{int.}}$) at Fe vacancies ($V_{\text{Fe}}$) in BCC Fe, and on simultaneous trapping of H and O atoms ($O_{\text{int.}}$). These configurations and their energetics are presented schematically in Figure 2a. It is known that a single Fe vacancy can trap up to six $H_{\text{int.}}$ atoms, which is correctly reproduced by the ACE potential. The presence of O in the $V_{\text{Fe}}-H_{\text{int.}}-O_{\text{int.}}$ cluster, reduces the favorable trapping to only four H atoms, likely due to chemical and steric effects of the nearby O atom. In both pristine vacancy and the $V_{\text{Fe}}-O_{\text{int.}}$ complex, the first H atom is trapped strongly, with an approximate segregation energy of −0.55 eV.

The strong binding between $H_{\text{int.}}$ atoms and $V_{\text{Fe}}$ affects the diffusion of both species, as reported in a number of previous studies [31, 35]. We investigated possible diffusion pathways and corresponding diffusion barriers for the coupled motion of a $V_{\text{Fe}}-H_{\text{int.}}$ complex. These results, showing intermittent trapping and de‐trapping of the H atom at the vacancy, are presented in Figure 2b.

Compared to the available DFT data [35], the ACE potential predicts a very close energy profile for such a complex diffusion path, involving bond breaking and forming [37]. We also include the predicted diffusion barriers for the pristine $V_{\text{Fe}}$ in BCC Fe (horizontal lines in Figure 2b). This barrier is higher than most intermediate steps of the coupled motion, but lower than the final step. This indicates that the influence of H on the diffusion properties of both species is rather complex.

#### 3.2.2 Hydrogen Diffusion

**FIGURE 3:**
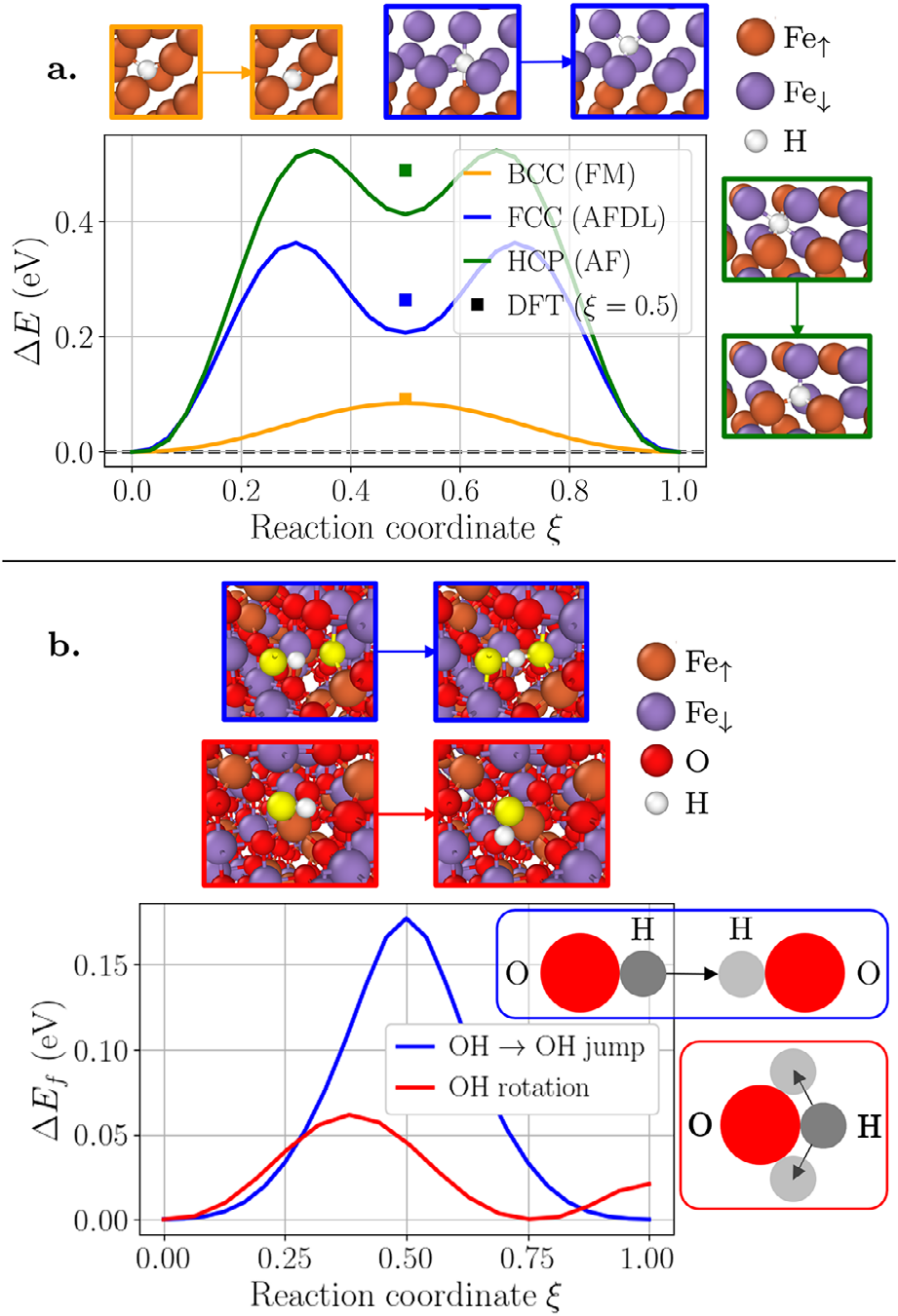
(a) Diffusion paths (at both ξ=0 and ξ=0.5) and associated energy barriers of a H interstitial atom in the three BCC (FM, orange), FCC (AFDL, blue) and HCP (AF, green) polymorphs of pure Fe, with DFT data at ξ=0.5 (squares). (b) Diffusion of a $H_{\text{int.}}$ atom in ${\mathrm{Fe}}_{3}O_{4}$: OH → OH jump (blue) and OH rotation (red). Atomic configurations with associated energy barriers for the two paths are discussed in the text.

We now examine the elementary diffusion pathways of interstitial H atoms in the BCC, FCC and HCP polymorphs of pure Fe. Under ambient conditions, only the FM BCC phase of pure Fe is stable. However, at high temperatures or pressures (e.g. under earth‐core conditions), both the FCC and HCP phases may also become stable. We thus test the ability of the ACE potential to capture the diffusion of $H_{\text{int.}}$ in these two phases as well. The lowest energy diffusion paths and associated energy barriers are presented in Figure 3a.

Among the three polymorphs, the diffusion of $H_{\text{int.}}$ atoms is fastest in the BCC phase, intermediate in the FCC phase, and slowest in the HCP phase, by order of increasing energy barrier opposing their diffusion. In the FCC and HCP phases, $H_{\text{int.}}$ atoms pass through a metastable tetrahedral site, (see Figure 3a).

We also studied the elementary diffusion pathways for $H_{\text{int.}}$ in the ${\mathrm{Fe}}_{3}O_{4}$ (see Figure 3b) and ${\mathrm{Fe}}_{2}O_{3}$ iron oxides. In both oxides, the diffusion path follows a two‐step mechanism: (1) the $H_{\text{int.}}$ rotates around the O atom of the oxide at which it is anchored, which has a low activation barrier; (2) when the $H_{\text{int.}}$ is aligned in the position of forming a new O–H bond with a neighboring O atom, it jumps to this new position, which is the rate‐limiting step of the diffusion process. In ${\mathrm{Fe}}_{3}O_{4}$, given the cubic symmetry of its O sub‐lattice, such jumps are equivalent in most directions of the lattice, whereas in ${\mathrm{Fe}}_{2}O_{3}$, since it has a HCP O sub‐lattice, jumps out of the basal {0001} planes, in which the initial O–H bonds are formed, are more favorable than in‐plane basal jumps.

### 3.3 Solute Interaction With Extended Defects

In this section, we examine on the interaction of interstitial H and O atoms with extended defects in pure Fe, focusing on dislocations and grain boundaries (GB) in the FM BCC phase.

#### 3.3.1 Grain Boundaries in BCC Fe

Grain boundaries are present in all polycrystalline materials and affect a wide range of materials properties, from diffusion to mechanical behavior [4, 5, 30, 38]. They usually act as strong traps for interstitial H atoms due to their disordered atomic environments and local regions of excess volume [33, 39]. Here, we inspect segregation tendencies of both O and H interstitial atoms to various GBs in the FM BCC phase of Fe. These calculations are of interest to rationalize the influence of the segregated elements on GB cohesion and intergranular cracking. A detailed comparison of formation energies for clean GBs obtained by ACE and DFT is provided in the Supporting Information.

**FIGURE 4:**
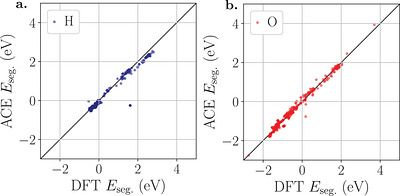
Correlation plots showing ACE vs. DFT‐predicted segregation energies $E_{\text{seg.}}$ at various BCC Fe GBs for H (a) and O (b) atoms. DFT references are taken from Ref. [33].

Figure 4 shows an excellent agreement between ACE predictions and DFT results [33] for segregation energies at six prototypical GBs. The ACE potential can closely reproduce the range of segregation energies spanning several eV for both H and O. The mean average errors for approximately 600 computed segregation energies for each element amount to 93 and 85 meV for O and H atoms, respectively. Such an agreement is indicative of the transferability and accuracy of the model, since very few GB configurations with segregated atoms were included in the training set of the ACE potential. Minimum segregation energies across the dataset of Ref. [33] are −2.76 and −0.54 eV for O and H interstitial atoms, respectively, confirming the existence of very strong trapping sites for both interstitial atoms in BCC Fe.

#### 3.3.2 Dislocations in BCC Fe

Dislocations are line defects governing plastic deformation that also act as favorable trapping sites for H and O [40, 41, 42, 43]. We investigated dislocations of different character (screw, edge and mixed) with two shortest Burgers vectors in BCC Fe, namely, 1/2⟨111⟩ and ⟨100⟩. The presence of interstitial atoms in the vicinity of the dislocation core strongly affects its mobility [40, 44, 45], and consequently the mechanical properties of the material.

For a fully decorated 1/2<111> screw dislocation line with one interstitial atom per unit line length (details of the dislocation simulations [46] are provided in the Supporting Information), we obtained interaction energies of −0.60 and −0.30 eV for O and H interstitials, respectively, comparing well with available DFT data of −0.52 eV [47] and −0.23 eV [32]. Both H and O atoms are referenced to their molecular $H_{2}$ and $O_{2}$ forms. Since the H atoms occupy a much smaller volume than the O atoms, three H atoms can segregate to the 1/2<111> screw dislocation core per unit‐length of the line, in contrast to only one O atom [32, 47]. Such an effect is also expected for other line orientations, as well as dislocations with a <100> Burgers vector.

For dislocations with other characters, we also considered two mixed orientation ($\vec{l}=\left[11\bar{1}\right]$ for $\vec{b}=1/2\left[111\right]$ and $\vec{l}=\left[11\bar{1}\right]$ for $\vec{b}=\left[100\right]$) and two edge orientations ($\vec{l}=\left[11\bar{2}\right]$ for $\vec{b}=1/2\left[111\right]$ and $\vec{l}=\left[0\bar{1}1\right]$ for $\vec{b}=\left[100\right]$). All these dislocations are glissile in {110} planes, i.e. both their Burgers vector $\vec{b}$ and their line direction $\vec{l}$ are contained in this plane. The segregation simulations for these dislocations were performed assuming the dilute limit of the segregating elements. The simulation cells had a line length of at least 7 Å to limit interactions between periodic replicas of the segregated atoms along the dislocation line. Starting from the relaxed ground state configuration of each dislocation core, we randomly placed one O or H atom in its vicinity and computed the segregation energy.

**FIGURE 5:**
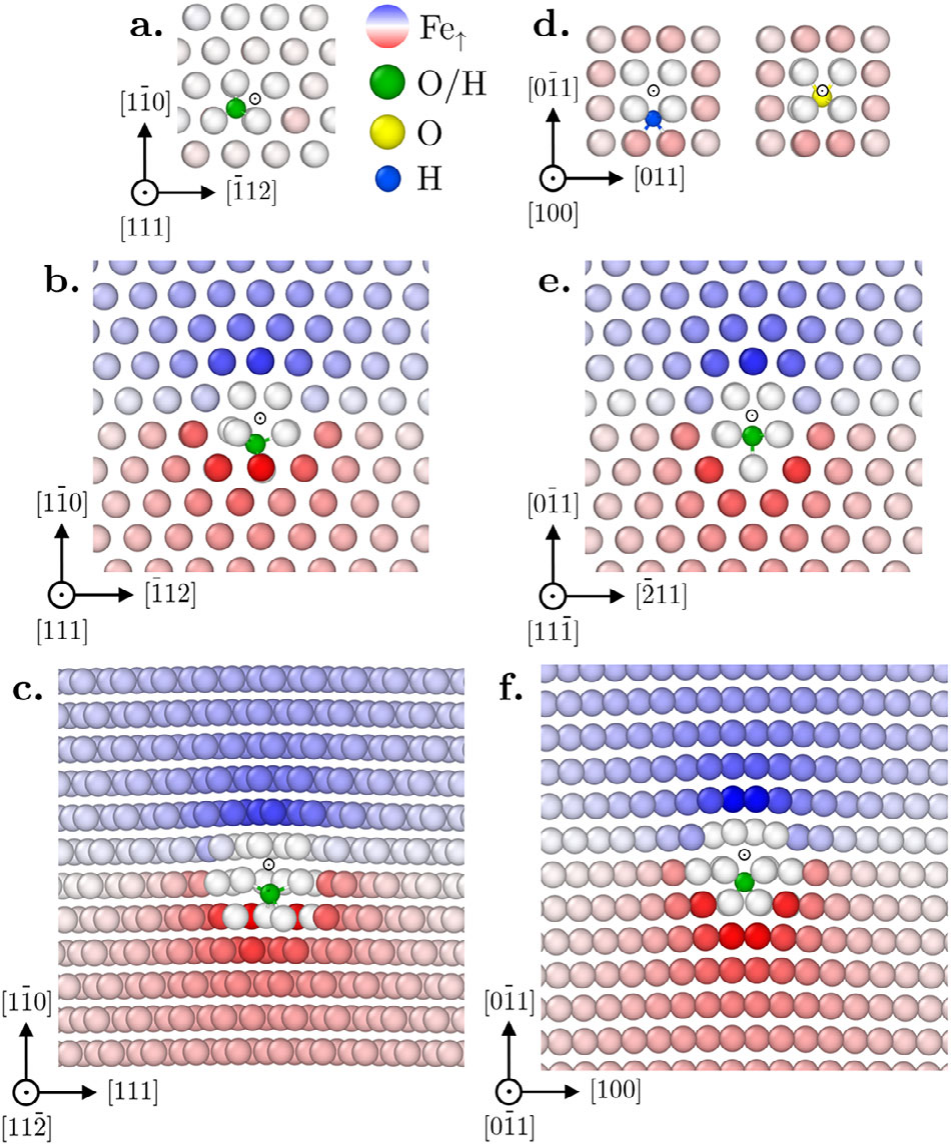
Core structures of (a–c) 1/2[111] and (d–f) [100] dislocations with a segregated O or H atom, considering different characters for both Burgers vectors: screw (a,d), mixed (b,e), and edge (c,f). Only non‐equivalent segregated core structures are shown, i.e. when both O and H atoms locate at the same site, the corresponding configuration is presented only once, with O and/or H atoms represented in green. Otherwise, O and H atoms are in yellow and blue, respectively. Fe atoms are colored according to the volumetric strain computed with respect to a bulk BCC Fe reference. Blue (red) colors indicate compressive (tensile) strains.

The lowest energy configurations of all considered dislocations predicted by the ACE potential are presented in Figure 5 and the numerical values are listed in Table 3.

**TABLE 3:** Minimum segregation energies $E_{\text{seg.}}^{H}$ and $E_{\text{seg.}}^{O}$ between single H and O atoms, respectively, and dislocations of both 1/2<111> and <100> Burgers vectors of different characters defined by their line orientations $\vec{l}$, as predicted by the ACE potential and corresponding to configurations presented in Figure 5. When available, DFT references (obtained using a different simulation setup) are presented in parenthesis.

	$E_{\text{seg.}}^{H}$ (eV)	$E_{\text{seg.}}^{O}$ (eV)
**Burgers vector** $\vec{b}=1/2\left[111\right]$		
Screw ($\vec{l}=\left[111\right]$)	−0.29 (−0.23 [32])	−0.76 (−0.52 [47])
Mixed ($\vec{l}=\left[11\bar{1}\right]$)	−0.39 (−0.37 [41])	−1.21
Edge ($\vec{l}=\left[11\bar{2}\right]$)	−0.41 (−0.37 [48])	−1.29
**Burgers vector** $\vec{b}=\left[100\right]$		
Screw ($\vec{l}=\left[100\right]$)	−0.49	−1.19
Mixed ($\vec{l}=\left[11\bar{1}\right]$)	−0.52	−1.44
Edge ($\vec{l}=\left[0\bar{1}1\right]$)	−0.56	−1.85

For the screw orientation of both dislocations (see Figure 5a,d), O and H atoms preferentially segregate at or near the core center. For the non‐screw orientations, the preferred segregation sites are located in the tensile region of the strain field induced by the dislocation, indicated by red Fe atoms in Figure 5. Overall, much stronger segregation tendencies are predicted by the ACE potential for the non‐screw dislocation cores (see Table 3).

The predictions of the ACE potential are again fully consistent with the available DFT data. For H segregating at the edge 1/2<111> dislocation, the DFT segregation energy of −0.37 eV [48], (using a rather small simulation cell) compares well with −0.41 eV obtained using the ACE potential. According to DFT, both H and O trigger a reconstruction of the 1/2<111> screw core from its equilibrium “easy” core configuration to the “hard” core configuration [32, 47]. As presented in the Supporting Information, the ACE potential reproduces this reconstruction as well.

When comparing the segregation tendencies presented in Table 3, we note that H atoms preferentially segregate to the <100> dislocations. As for O atoms, segregation to the <100> dislocations is also stronger than to the 1/2[111] dislocations, but the edge orientation of the latter type shows a stronger segregation tendency than the screw orientation of the former, in contrast with the behavior of H atoms.

Overall, the strongest GB trapping sites (minimum segregation energies of −2.76 and −0.54 eV for O and H, respectively) are comparable to the strongest dislocation trapping sites (minimum segregation energies of −1.85 and −0.56 eV for O and H interstitial atoms, respectively), albeit O shows weaker affinity to dislocations than to GBs. Nevertheless, both families of these extended defects represent stronger traps than single vacancies in BCC Fe (*cf*. Figure 2).

#### 3.3.3 BCC Fe Surfaces

Lastly, we focus on the interaction of H and O with various surface facets of BCC Fe. Surfaces represent the first contact point between any liquid or gas environment with the bulk of the material and their atomic structure and chemical composition are therefore of high relevance [49]. The interactions with common ambient species, such as H and O, can affect the relative stability of different surface orientations and induce their reconstructions due to formation of an oxide surface layer [50] or accumulation of adsorbed species [51]. Even small amounts of H and O can lead to surface contamination [52] that is remarkably resilient and can persist even under ultra high vacuum conditions.

**FIGURE 6:**
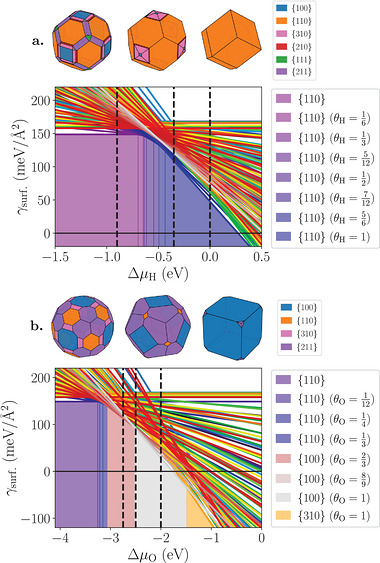
Surface phase diagrams of BCC Fe surfaces covered by H atoms (a), and O atoms (b), as a function of the H/O chemical potential change $\Delta \mu_{\text{H/O}}$ with respect to molecular $H_{2}$/$O_{2}$. For each surface phase diagram, we present Wulff constructions of equilibrium nanoparticle morphologies (plotted using the wulffpack package [53]) at the three $\Delta \mu_{\text{H/O}}$ values indicated by black vertical dashed lines on both surface phase diagrams. Only labels of stable surface coverages are presented in the legends. For more detailed facet‐specific surface phase diagrams, please refer to the Supporting Information.

In this section, we present the coverage‐dependent adsorption energies of single H and O atoms on various surfaces of BCC Fe. The simulations were carried out for a number of different random configurations of surface O and H atoms placed on various surfaces of BCC Fe (namely {100},{110},{111},{210},{211},{221},{310},{320} and {321}) with different coverages. Based on the lowest energy configurations for each surface coverage and for each surface orientation, we built chemical‐potential‐dependent surface phase diagrams for both O and H adsorbates, which are presented in Figure 6.

For H adsorbates (see Figure 6a), we observed a stabilization of the {110} facets with increasing surface coverage as $\mu_{H}$ increases. We also computed the equilibrium Wulff shapes under various chemical conditions, using the wulffpack package [53]. Such a stabilization of {110} surfaces with the increasing H chemical potential leads to Wulff shapes exposing only the {110} facets for $\Delta \mu_{H}>-0.1$ eV (see upper panel in Figure 6a). Experimental data based on desorption energies [49, 54] as well as DFT calculations [55] support such an observation. Given the close agreement between our ACE predictions, the DFT results published by Wang et al. [55], and our present DFT data (see Table 4 for individual H adsorption energies of various surface facets), we expect similarly good agreement with experiment.

**TABLE 4:** Adsorption energy $E_{\text{ads.}}^{H}$ (in eV) of a single H atom on different surface facets of BCC Fe, as predicted by the ACE potential and DFT (GGA‐PBE) calculations (this work, and Wang et al. [55]). No ZPE correction is included.

Facet	ACE	DFT
{100}	−0.62	−0.36, −0.46 [55]
{110}	−0.70	−0.71, −0.70 [55]
{111}	−0.59	−0.59, −0.51 [55]
{210}	−0.69	−0.68, −0.66 [55]
{211}	−0.64	−0.51, −0.60 [55]
{221}	−0.67	/
{310}	−0.68	−0.63, −0.65 [55]
{311}	−0.68	/
{320}	−0.70	/
{321}	−0.68	−0.65 [55]

As for O adsorbates (see Figure 6b), the picture differs from H adsorbates, with a stabilization of different surface orientations across the range of $\mu_{O}$ scanned. As is also reflected on the computed Wulff equilibrium shapes (see upper panel in Figure 6b), the stability of {110} facets at low $\mu_{O}$ is lifted in favor of an increasing fraction of both {211} and {100} facets at higher $\mu_{O}$, until {100} facets dominate under high equivalent oxygen pressures. In both cases (i.e. considering either O or H adsorbates), we predict much sharper equilibrium Wulff morphologies compared to the one obtained considering clean surfaces, with one or two different predominant surface facets exposed under high chemical potential of either H or O.

We also present in Supporting Information coverage‐dependent adsorption energies of both O and H atoms on various surfaces of BCC Fe, showing different interaction behaviors between adsorbates depending on the surface orientation.

### 3.4 Probing Atomistic Processes With Molecular Dynamics Simulations

We now asses the transferability of the ACE potential by performing atomistic simulations of complex processes such as hydrogen‐driven reduction of iron oxide surfaces, H permeation into Fe surfaces, accelerated pipe and GB diffusion, and the interaction of water with metallic iron surfaces. We additionally present in Supporting Information, Note S5, properties of iron‐oxygen liquids, comparing predictions of the ACE potential with various reaxFF parameterizations and experimental data, as well as the near‐melting and dehydroxylation of a FeO(OH) sample along a MD simulation.

#### 3.4.1 Hydrogen Reduction of a ${\mathrm{Fe}}_{2}O_{3}$ Surface

Hydrogen‐based direct reduction of iron oxides, a process envisioned to mitigate the ${\mathrm{CO}}_{2}$ footprint of the steelmaking industry [3], involves a wide range of chemical reactions, phase transformations, and transport phenomena [57, 58, 59, 60], which govern the kinetics of the whole process. Most of the critical mechanisms occur at the atomic scale [57], which is not easily accessible to experiments, but which can be conveniently probed by large‐scale atomistic simulations.

**FIGURE 7:**
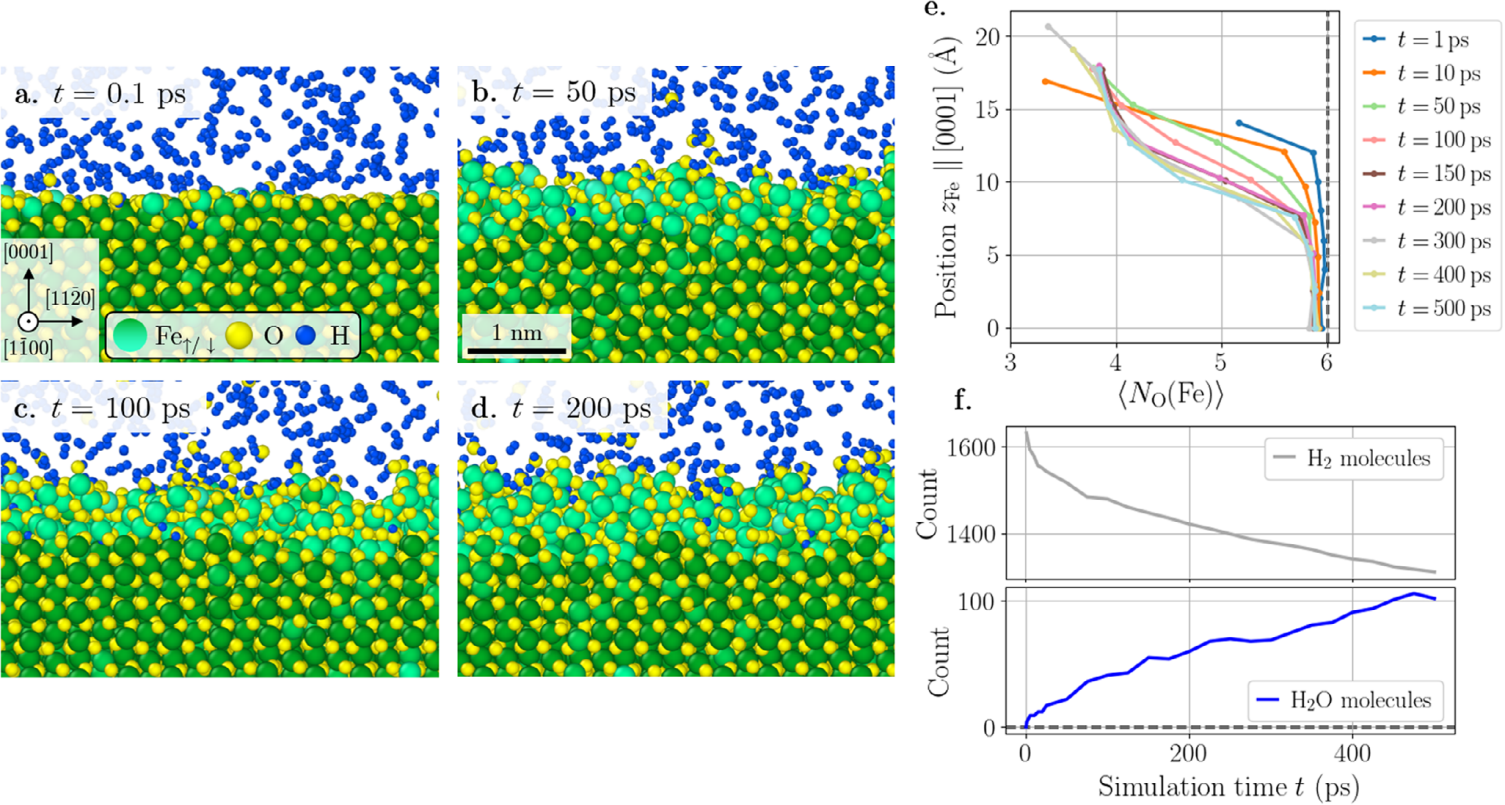
(a–d) Snapshots at different time of a molecular dynamics simulation of a ${\mathrm{Fe}}_{2}O_{3}$ slab exposing two identical O‐rich (0001) basal surfaces to a hydrogen $H_{2}$ gas at 1000 K. On each snapshots, Fe atoms are colored as a function of the number of their O neighbors, from light green (less than two neighbors) to green (six neighbors, i.e. the pristine bulk coordination). Not all of the simulation cell is shown, focusing for clarity on the top surface, and considering only a 40 Å‐thick slice along the $1\bar{1}00$ direction. O and H atoms are represented in yellow and blue, respectively. The ovito package [56] was used for visualization. (e) Depth profiles at different simulation times t showing the average coordination of Fe atoms in terms of O neighbors $\left\langle N_{O}\left(\text{Fe}\right)\right\rangle$ through the ${\mathrm{Fe}}_{2}O_{3}$ slab along the [0001] direction. The profiles from the two surfaces of the slab are averaged before plotting. (f) Evolution of the molecules in the gas phase ($H_{2}$ and $H_{2}O$) as a function of simulation time t.

In this section, we provide atomistic insights into the interactions of $H_{2}$ molecular gas with pristine iron oxide surfaces, which represent the very first stages of the reduction process. In the simulations, we expose a slab of pristine ${\mathrm{Fe}}_{2}O_{3}$ with O‐rich terminated {0001} surfaces to a $H_{2}$ atmosphere with an approximate initial partial pressure of $P_{H_{2}}=1$ kbar. The O‐rich {0001} surface of ${\mathrm{Fe}}_{2}O_{3}$ has been extensively studied experimentally [61, 62, 63, 64] and presents thus a convenient system for a comparison between theoretical predictions and experimental findings. The system is then heated to 1000 K under NPT conditions (the barostat only applies to in‐plane dimensions of the cell) and its dynamical evolution is observed for 500 ps. Snapshots of the MD trajectory are presented in Figure 7a–d at different times of the simulation.

During the MD simulation, we observe a variety of different surface and bulk phenomena, starting from the formation of water ($H_{2}O$) molecules, the elementary chemical reaction on which the whole hydrogen‐based reduction process relies. Following the removal of O atoms from the ${\mathrm{Fe}}_{2}O_{3}$ slab, occurring at the interface with the reducing $H_{2}$ atmosphere, we measure the progressive reduction of the material by tracking the number of O neighbors of each Fe atom. We visualize such a reduction in Figure 7a–d by coloring Fe atoms by decreasing shades of green as the number of their O neighbors decreases, an indicative measure of the local progress of the reduction reaction.

We further quantify such a reduction in Figure 7e, where the average number of O neighbors $\left\langle N_{O}\left(\text{Fe}\right)\right\rangle$ is plotted as a function of the depth into the ${\mathrm{Fe}}_{2}O_{3}$ slab at different times along the MD trajectory. We observe a quick reduction from the outermost surfaces exposed to the $H_{2}$ gas, within the first picoseconds of the simulation. As the reaction proceeds, more $H_{2}O$ molecules are formed, which leads to a gradual reduction of the number of $H_{2}$ molecules in the gas phase (*cf*. Figure 7f). The average $\left\langle N_{O}\left(\text{Fe}\right)\right\rangle$ for Fe atoms close to the surface also gradually decreases, leaving only the bulk part of the slab with six‐fold coordinated Fe atoms of the equilibrium ${\mathrm{Fe}}_{2}O_{3}$ structure.

The exchange layer, defined as the region where Fe atoms are being reduced, extends over a depth of approximately 1 nm after a few hundreds of picoseconds (see Figure 7e). Additionally, we observe a drastic change in the morphology of the surface as the reaction proceeds. The crystal structure of the exchange layer becomes rather disordered, which affects the kinetics of the reduction process. Initially, O atoms participating in the reduction reaction are readily available at the exposed, O‐rich top surface layer, explaining the sharp increase of the $H_{2}O$ molecules formed and decrease of the $H_{2}$ molecules at the very beginning of the simulation (see Figure 7f). For the reduction process to continue, more O atoms need to come into contact with the $H_{2}$ gas, and thus must diffuse from bulk ${\mathrm{Fe}}_{2}O_{3}$ to the surface region. This second stage, starting after approximately 50 ps, is therefore characterized by a slower formation rate of $H_{2}O$ molecules. In the current setup, as the simulation progresses, the chemical potential driving force for reducing the oxide also decreases, since the gas phase contains less $H_{2}$ molecules, influencing the process along the simulation.

A variety of complex atomistic phenomena were uncovered during the presented MD simulation, which enable us to rationalize the kinetics and thermodynamic driving forces behind the experimental process. Here, we present preliminary results, while more details and analysis will be discussed in a follow‐up publication focusing solely on the hydrogen‐driven reduction of iron oxides.

#### 3.4.2 Hydrogen Surface Permeation and Diffusion

The ingress of H into bulk consists of adsorption of $H_{2}$ molecules on the surface, their subsequent dissociation, and the permeation and diffusion of single H atoms into the microstructure [30, 31].

**FIGURE 8:**
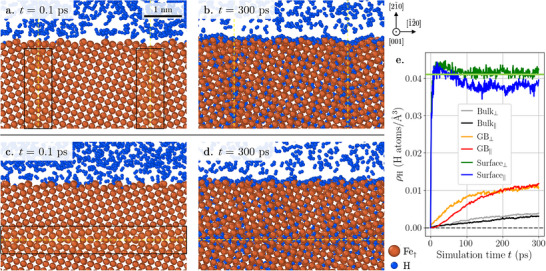
(a–d) Snapshots of two molecular dynamics simulations of a BCC Fe slab containing $\Sigma 5\left[001\right]\left(2\bar{1}0\right)$ GBs in contact with $H_{2}$ gas, for a setup with the GB planes (yellow dashed lines) perpendicular (a,b) and parallel (c,d) to the surface, at 0.1 ps (a,c) and 300 ps (b,d). Only part of the whole simulation cell is shown for clarity. Fe and H atoms are represented in orange and blue, respectively. (e). Evolution of H volume density $\rho_{H}$ as a function of simulation time t in the three regions of the simulation cells (bulk, GB and surface), for both setups, i.e. GB planes perpendicular (⊥) and parallel (∥) to the surfaces exposed to hydrogen gas. Regions for the evaluation of $\rho_{H}$ at GBs are indicated by black shaded boxes on (a,c). The horizontal light green line indicates the predicted equilibrium surface H coverage $\theta_{H}$ of {210} surfaces obtained from surface phase diagrams presented in Figure 6 (see also Supporting Information for the {210} surface phase diagram) under the conditions of the MD simulation ($P_{H_{2}}\approx 1$ kbar and T=1000 K). The ovito package [56] was used for visualization.

We simulated these processes for BCC Fe bi‐crystals terminated by the {210} surfaces. The bi‐crystals were separated by a $\Sigma 5\left[001\right]\left(2\bar{1}0\right)$ GBs, which was investigated in several studies of H segregation [33, 41, 44]. To examine the influence of GB orientation on the H permeation, we employed two simulation geometries: one with the GB planes (there are two GBs present due to periodic boundary conditions applied in the simulations) oriented perpendicular to the surface (⊥, Figure 8a,b); and another one with the GB located about 2 nm below the surface and oriented parallel to the surface plane (∥, Figure 8c,d). The vacuum region above the free surfaces was filled with $H_{2}$ molecules, at a pressure of 1 kbar. The evolution of the system was simulated at 1000 K for 300 ps.

For both systems, we observed a very fast adsorption of the $H_{2}$ molecules onto the surface and their subsequent dissociation. Once the $H_{2}$ bond is broken, H atoms start diffusing into the bulk. To analyze the diffusion through the simulation cell, we defined three regions of interest: bulk, GBs and surfaces. The volume density of H atoms $\rho_{H}$ as a function of time in these three regions is presented in Figure 8e.

First, one can see the rapid population of surfaces with H within the first few ps of the simulations that remains almost constant afterward. The horizontal green line in the graph indicates the equilibrium surface H coverage $\theta_{H}$ predicted from surface phase diagrams presented in Figure 6a (see also Supporting Information for the {210} surface phase diagram of BCC Fe, as well as other surface orientations). The predicted value for $\theta_{H}$ matches well the equilibrium reached during the MD simulations, showing a consistent picture from thermodynamic analysis and direct MD simulations.

Second, the MD simulations reveal that H permeates faster in the system with the perpendicular GBs terminating at the surface than in the system with the parallel GB below the surface. At the elevated temperature of the simulation, this suggests that the $\Sigma 5\left[001\right]\left(2\bar{1}0\right)$ GB thus acts as an easy channel for H diffusion. This observation is quantified by time‐dependent segregation profiles of H at the GBs in Figure 8e, which can be rationalized by various phenomena, in particular the balance between diffusion and trapping of interstitial H atoms at GBs.

Finally, the H concentration in the bulk region increases at the slowest pace. This does not necessarily indicate that the bulk diffusion is slow, since the migration barrier for H diffusion in BCC Fe is very low (*cf*. Figure 3a). This is confirmed by quick filling of the parallel GB located below the surface with H. The overall apparent diffusion and distribution of H throughout the system is therefore governed by different H mobilities in different regions, as well as trapping at GBs and surfaces, which requires a careful evaluation of both aspects to be fully rationalized [44].

As compared to the pristine ${\mathrm{Fe}}_{2}O_{3}$ slab in contact with high temperature and pressure $H_{2}$ gas in the previous section, we note that the dissociation of $H_{2}$ molecules on the surface is much more effective when metallic Fe is in contact with the hydrogen gas. Such an effect could catalyze the dissociation of $H_{2}$ molecules in the context of hydrogen‐based reduction of iron oxides, and will be addressed in more details in a follow‐up publication.

#### 3.4.3 BCC Fe Surface Reactions With Water

**FIGURE 9:**
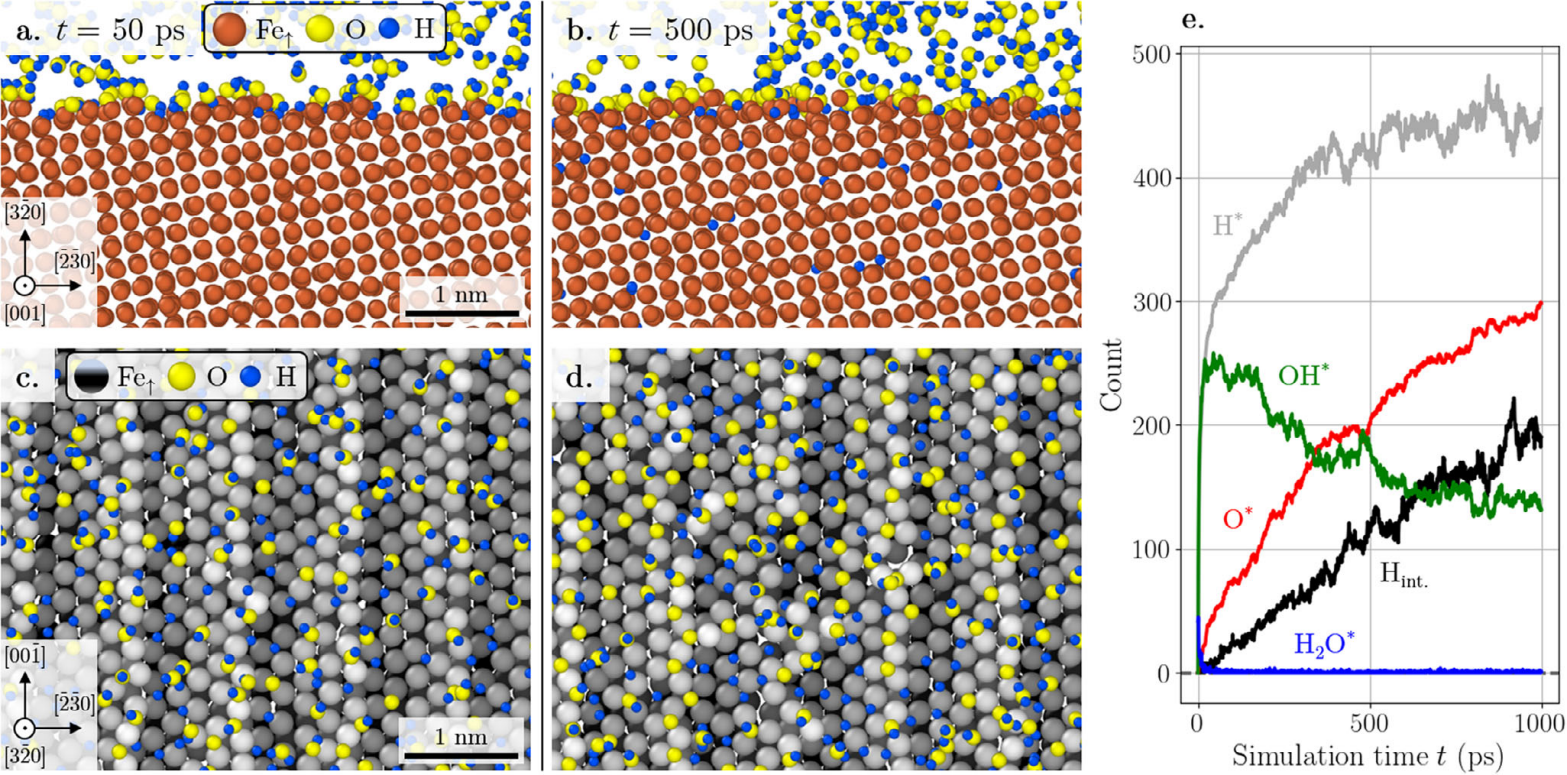
(a–d) Snapshots from the MD simulation of a BCC Fe slab terminated by $\left(3\bar{2}0\right)$ surfaces reacting with water vapor of density $\rho_{H_{2}O}=0.40$ g/${\mathrm{cm}}^{3}$ at 1000 K. Side (a,b) and top (c,d) views of the system at 50 ps (a–c) and 500 ps (b–d). Only part of the whole simulation cell is shown for clarity. In the top views, Fe atoms are colored as a function of their heights along the $\left[3\bar{2}0\right]$ direction to highlight the position of surface terraces, from black (bottom) to gray (top). In the side views (a,b), only a 20 Å‐thick slice of the full simulation cell along the [001] direction is shown. Otherwise, Fe, O and H atoms are represented in orange, yellow and blue, respectively. (e). Evolution of the number of surface adsorbed species (H, O, OH and $H_{2}O$ with a superscript 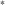) and H atoms in the bulk ($H_{\text{int.}}$) as a function of simulation time t. The ovito package [56] was used for visualization.

The corrosion of ferrous alloys represents a great technological challenge, as it leads to spontaneous degradation and life‐time reduction of these materials. In the context of water vapor corrosion and oxidation of metallic Fe, we performed an MD simulation of the interaction of water vapor of initial density $\rho_{H_{2}O}=0.40$ g/${\mathrm{cm}}^{3}$ with a {320} surface of BCC Fe at a temperature of 1000 K. Snapshots from the simulation are presented in Figure 9a–d. To better visualize surface sites and reactions, Fe atoms in the top views of Figure 9c,d are colored from black to white according to their proximity to the surface.

The primary process we focused on was the dissociation of $H_{2}O$ molecules on the surface. We observed that the water molecules dissociate immediately after they attach to the Fe surface atoms with the most active sites for the reaction located at the edges of the {110} facets of the {320} surface. To quantify the evolution of the reaction products, we analyzed the population of surface species as a function of simulation time, as presented in Figure 9e. The graph shows an abrupt increase of ${\text{OH}}^{*}$ groups and $H^{*}$ atoms within the first few ps of the simulations that corresponds to the initial adsorption of water molecules to the pristine Fe surface. The equal amounts of both products (up to count of about 250) indicate that almost every $H_{2}O$ molecule landing on the surface dissociates according to $H_{2}O\to {\text{OH}}^{*}+H^{*}$.

After this initial stage at approximately 50 ps of the simulation, the population of the ${\text{OH}}^{*}$ surface groups saturates and starts to decrease. This change coincides with an increase of H atoms in the bulk region of the slab ($H_{\text{int.}}$ marked by black curve) and individual O atoms at the surface ($O^{*}$ marked by red curve). Therefore, the ${\text{OH}}^{*}$ surface groups undergo subsequent dissociations into $O^{*}$ and $H^{*}$ or $O^{*}$ and $H_{\text{int.}}$, which are maintained throughout the whole simulation.

The diffusion of H atoms into the bulk is consistent with the previous simulation presented above and results from the difference of H chemical potential in different regions of the slab. Absence of O atoms in the slab is likely related to a much higher migration barrier of interstitial O atoms in bulk BCC Fe.

Finally, we also observe the formation of $H_{2}$ and $H_{3}O$ molecules in the gas phase due to desorption of H atoms from the surface. Due to short simulation time and high temperature, no considerable oxide layer is formed at the end of the MD simulation, but we can see a disappearance of the surface facets and increased disorder of the surface layer.

## 4 Conclusion

In this work, we developed an ACE potential for the ternary Fe‐O‐H system, with an explicit account of magnetism in an efficient Ising‐like manner. We demonstrated the ability of the model to describe fundamental properties of various materials encompassed by the system, from pure iron to iron oxides, hydrides and hydroxide, including defects and magnetic effects.

We further demonstrated the transferability of the developed ACE potential by directly simulating various atomistic mechanisms taking place during hydrogen‐based reduction of iron oxides, permeation and diffusion of hydrogen into surfaces and through grain boundaries in iron, and corrosion of metallic iron surfaces by water vapor. Such a versatile ACE model will then allow to directly probe and analyze atomistic mechanisms underlying those various processes, in an attempt to rationalize experimental observations through well‐designed targeted atomistic simulations.

One of the main remaining improvements of the model would be an explicit account of charges [65], which are of great physical relevance in iron oxides and various processes, and are not described by the present ACE potential, also because the DFT xc‐functional (GGA‐PBE) used to train the model predicts most materials within the Fe‐O system to be metallic. We also stress that our choice for the DFT flavor used for training the model might yield shortcomings in some regions of the ternary Fe‐O‐H system (e.g. chemical reactions and charge‐related phenomena). However, such effects are difficult to predict, and further comparison with experimental references should allow to assess the description of these phenomena by the present ACE potential. Another possible improvement of the potential would be to describe magnetic degrees of freedom using a more complex model, for instance including longitudinal excitations in a collinear model, or even a non‐collinear description of magnetic moments, as recently achieved in elemental iron within the ACE formalism [13].

## Conflicts of Interest

The authors declare no conflicts of interest.

## Supporting information


**Supporting File**: advs76709‐sup‐0001‐SuppMat.pdf.

## Data Availability

Files for the Fe‐O‐H ternary ACE potential, as well as sample scripts and explanatory notes on its implementation in the lammps code [66], are available at https://zenodo.org/records/20793272.
