# Supplementary material for: Development and Applications of a Magnetism‐Aware General‐Purpose Atomic Cluster Expansion Potential for the Fe‐O‐H Ternary System
Source: Adv Sci (Weinh). 2026 Jul 27:e76709. Online ahead of print. doi: 10.1002/advs.76709 (PMC13404870; doi:10.1002/advs.76709)
Supplement: Supplementary file 1 — Supporting File: advs76709‐sup‐0001‐SuppMat.pdf. [file ADVS-9999-e76709-s001.pdf]

Supplementary Information: Development and applications of a magnetism-aware general-purpose Atomic Cluster Expansion potential for the Fe-O-H ternary system

Baptiste Bienvenu,* Mira Todorova,† Jörg Neugebauer,‡ and Dierk Raabe§

Max Planck Institute for Sustainable Materials,

Max-Planck-Straße 1, 40237 Düsseldorf.

Matous Mrovec and Ralf Drautz

Interdisciplinary Centre for Advanced Materials Simulations,

Ruhr Universität Bochum, 44780 Bochum.

*Electronic address: b.bienvenu@mpi-susmat.de

†Electronic address: m.todorova@mpi-susmat.de

‡Electronic address: j.neugebauer@mpi-susmat.de

§Electronic address: d.raabe@mpi-susmat.de

Table of content:

- Supplementary Note S1: Phonon spectra of FeH polymorphs and FeO(OH)
- Supplementary Note S2: Validation on the binary Fe-O subsystem
- Supplementary Note S3: Clean grain boundary energies in BCC Fe
- Supplementary Note S4: Impact of magnetic order on selected properties
- Supplementary Note S5: Properties of liquids and near-melting conditions
- Supplementary Note S6: Interaction energies between dislocation cores and interstitial O and H atoms in BCC Fe
- Supplementary Note S7: Surface-specific phase diagram of BCC Fe with adsorbed H and O atoms
- Supplementary Note S8: Simulation setups and details
- Supplementary Note S9: Structure of the DFT training set
- Supplementary Note S10: Model performance and limitations

Supplementary Note S1: Phonon spectra of FeH polymorphs and FeO(OH)

As additional validation of the potential, we present in Supplementary Figs. 1 and 2 the phonon spectra for the three FeH polymorphs (*i.e.* FCC, HCP and DHCP, all with a ferromagnetic order), and FeO(OH), with an antiferromagnetic order. All structures show no imaginary phonon mode, showing their dynamical stability predicted by the ACE potential.

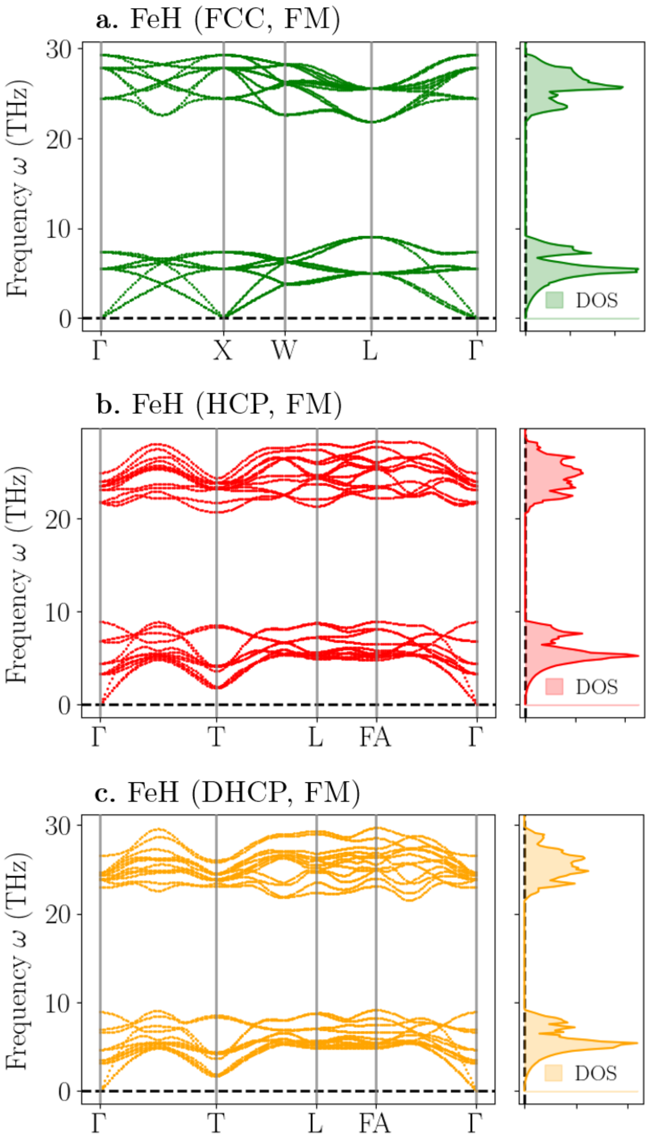

Supplementary Figure 1: Phonon spectrum (left) and total density of states (right) of (**a**) FCC FeH (FM order), (**b**) HCP FeH (FM order), and (**c**) DHCP FeH (FM order), as predicted by the ternary ACE potential.

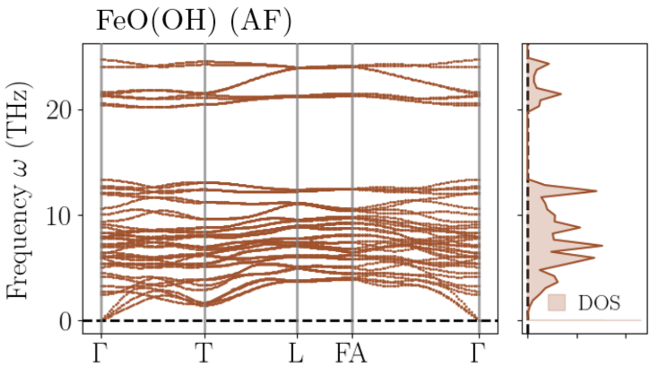

Supplementary Figure 2: Phonon spectrum (left) and total density of states (right) of FeO(OH) (AF order), as predicted by the ternary ACE potential.

For the FCC phase of FeH, a $2 \times 2 \times 2$ supercell with axis along the three [100], [010] and [001] directions was used. As for both HCP and DHCP phases of FeH, a $3 \times 3 \times 1$ supercell of the primitive hexagonal cell was used. For FeO(OH), a $3 \times 2 \times 1$ supercell of the conventional orthogonal cell was used.

Supplementary Note S2: Validation on the binary Fe-O subsystem

As discussed in the main text, since the parameters of the ternary Fe-O-H potential describing the binary Fe-O subsystem have been slightly adjusted during the fitting of the present potential, we validate and compare the predictions of the two ACE potentials, *i.e.* binary Fe-O from our previous work [1] and the present Fe-O-H parameterization, for properties covering the binary Fe-O subsystem.

Training errors

In this respect, we first compare the errors, in total energies and force components, committed by the two Fe-O and Fe-O-H ACE potentials on the DFT training set we used to parameterize the binary potential in our previous work [1]. For the binary Fe-O potential, we get a mean average error (MAE) of 22 meV/atom in energies, and 78 meV/Å in forces. As for the Fe-O-H ternary potential, we get a MAE of 25 meV/atom in energies, and 88 meV/Å in forces, showing how close the two potentials compare on the binary Fe-O training set.

Basic properties

We also compare predictions of the two Fe-O and Fe-O-H ACE potentials on bulk and simple defect (vacancy, self-interstitials and surfaces) properties of BCC Fe in Supplementary Tab. I, and bulk properties of the three stable oxides (*i.e.* FeO, Fe_3O_4 and Fe_2O_3) in Supplementary Tab. II. We also compared these predictions with DFT (GGA-PBE exchange-correlation functional) and available experimental data. We also present in Supplementary Fig. 3 energy-volume curves for Fe polymorphs (*i.e.* BCC, FCC, HCP and *A*15) and the three iron oxides, obtained with the two ACE potentials, also compared with DFT calculations. For these properties, we obtain a very good agreement between the two ACE potentials, as well as with DFT and experimental references, showing how the ternary Fe-O-H potential is as accurate as our previous model [1] across the binary Fe-O sub-system.

Supplementary Table I: Bulk properties (lattice parameter a_0, bulk modulus B_0 and elastic constants C_{ij}) of BCC Fe (FM order), and defect formation energies (vacancy $E_f^{\text{vac.}}$ also with the height of the diffusion barrier $\Delta E^{\text{vac.}}$, self-interstitial atom with various dumbbell configurations E_f^{dhkl}, and surface energies $\gamma^{\{hkl\}}$, where hkl denote the Miller indices of the crystallographic orientation). Properties are predicted by the binary Fe-O [1] and ternary Fe-O-H ACE potentials, and also compared to DFT and experiments.

	ACE Fe-O	ACE Fe-O-H	DFT	Expt.
Fe, BCC FM				
a_0 (Å)	2.836	2.831	2.834	2.860 [2]
B_0 (GPa)	165	177	191 [3]	170 [4]
C_{11} (GPa)	240	263	283 [3]	240 [4]
C_{12} (GPa)	127	134	145 [3]	136 [4]
C_{44} (GPa)	91	99	104 [3]	121 [4]
$E_f^{\text{vac.}}$ (eV)	2.09	2.06	2.20	2.0 ± 0.2 [5]
$\Delta E^{\text{vac.}}$ (eV)	0.67	0.70	0.70	/
E_f^{d110} (eV)	4.5	4.6	4.9 [3]	4.7 - 5.0 [6]
E_f^{d100} (eV)	5.4	5.7	5.3 [3]	/
E_f^{d111} (eV)	4.7	4.9	4.9 [3]	/
$\gamma^{\{110\}}$ (J/m^2)	2.52	2.39	2.42	/
$\gamma^{\{100\}}$ (J/m^2)	2.58	2.55	2.49	/
$\gamma^{\{112\}}$ (J/m^2)	2.71	2.66	2.56	/
$\gamma^{\{111\}}$ (J/m^2)	2.79	2.77	2.69	/

Supplementary Table II: Bulk properties (lattice parameter a_0, bulk modulus B_0), and formation enthalpy ΔH_f of the three FeO, Fe_3O_4 and Fe_2O_3 iron oxides. Properties are predicted by the binary Fe-O [1] and ternary Fe-O-H ACE potentials, and also compared to DFT (using the GGA-PBE exchange-correlation functional) and experiments.

	ACE Fe-O	ACE Fe-O-H	DFT	Expt.
Stoichiometric wüstite, FeO (distorted NaCl, AF)				
a_0 (Å)	4.29	4.30	4.26	4.33 [7]
B_0 (GPa)	181	174	195	175 [7]
ΔH_f (eV/atom)	−0.95	−0.95	−0.91	−1.41 [8]
Magnetite, Fe_3O_4 (inverse spinel, FeM)				
a_0 (Å)	8.40	8.40	8.40	8.40 [9]
B_0 (GPa)	165	160	172	185 [9]
ΔH_f (eV/atom)	−1.29	−1.29	−1.29	−1.66 [8]
Hematite, Fe_2O_3 (corundum, AF)				
a (Å)	4.98	4.99	5.02	5.04 [10]
c (Å)	14.06	13.97	13.90	13.75 [10]
B_0 (GPa)	156	159	172	225 [10]
ΔH_f (eV/atom)	−1.30	−1.30	−1.30	−1.71 [8]

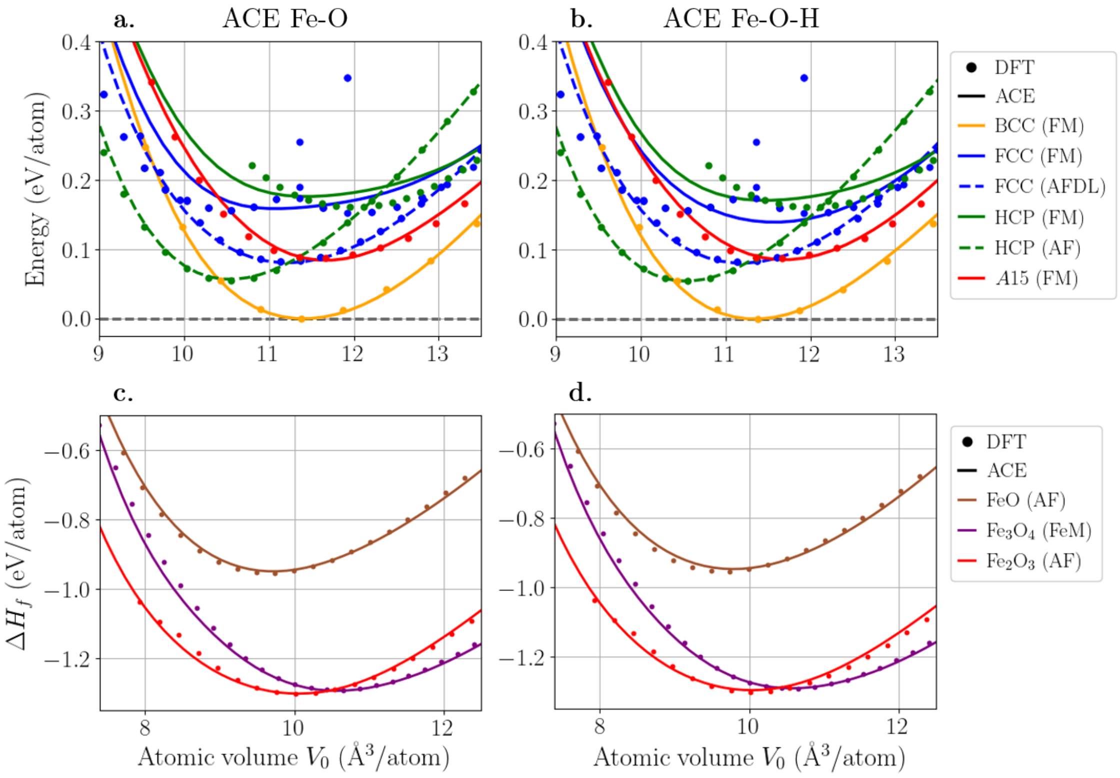

Supplementary Figure 3: Energy-volume curves for (**a-b**) Fe polymorphs, and (**c-d**) Fe oxides, as predicted by the binary Fe-O (left column) and ternary Fe-O-H (right column) ACE potentials. DFT data are plotted as symbols.

Defect properties

Additionally, we present in Supplementary Fig. 4 diffusion barriers of an interstitial O atom in BCC Fe, in the bulk matrix, and in the vicinity of an Fe vacancy, as obtained with the two ACE potentials, also showing close predictions between the two models.

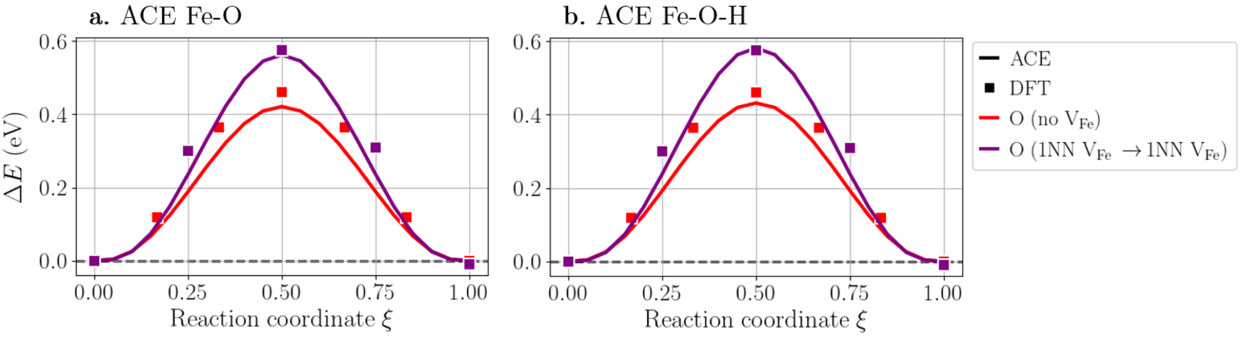

Supplementary Figure 4: Interstitial oxygen diffusion in BCC Fe (FM magnetic order) in the bulk matrix (red) and in the vicinity of a vacancy (purple), obtained with the (**a**) Fe-O and (**b**) Fe-O-H ACE potentials, and also compared to DFT calculations (symbols).

We also compare predictions of the two ACE models regarding the core structure and the Peierls energy barrier opposing glide of a $1/2\langle 111 \rangle$ screw dislocation in a $\{110\}$ plane in BCC Fe, presented in Supplementary Fig. 5.

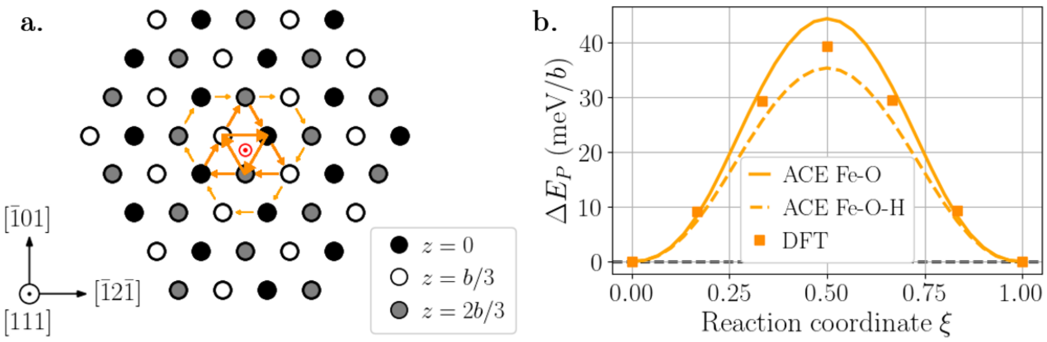

Supplementary Figure 5: (**a**) Core structure of a $1/2\langle 111 \rangle$ screw dislocation, predicted by the two binary and ternary ACE potentials plotted as a differential displacement map along the [111] direction. (**b**) Peierls energy barrier opposing glide of a $1/2\langle 111 \rangle$ screw dislocation in a $\{110\}$ plane between two adjacent equilibrium positions, as predicted by the two ACE potentials and compared to DFT data from Ref. [11].

Diffusion barriers of Fe and O vacancies in the two Fe_3O_4 and Fe_2O_3 iron oxides, as

predicted by the two models, are presented in Supplementary Fig. 6, also showing close predictions for all presented diffusion barriers.

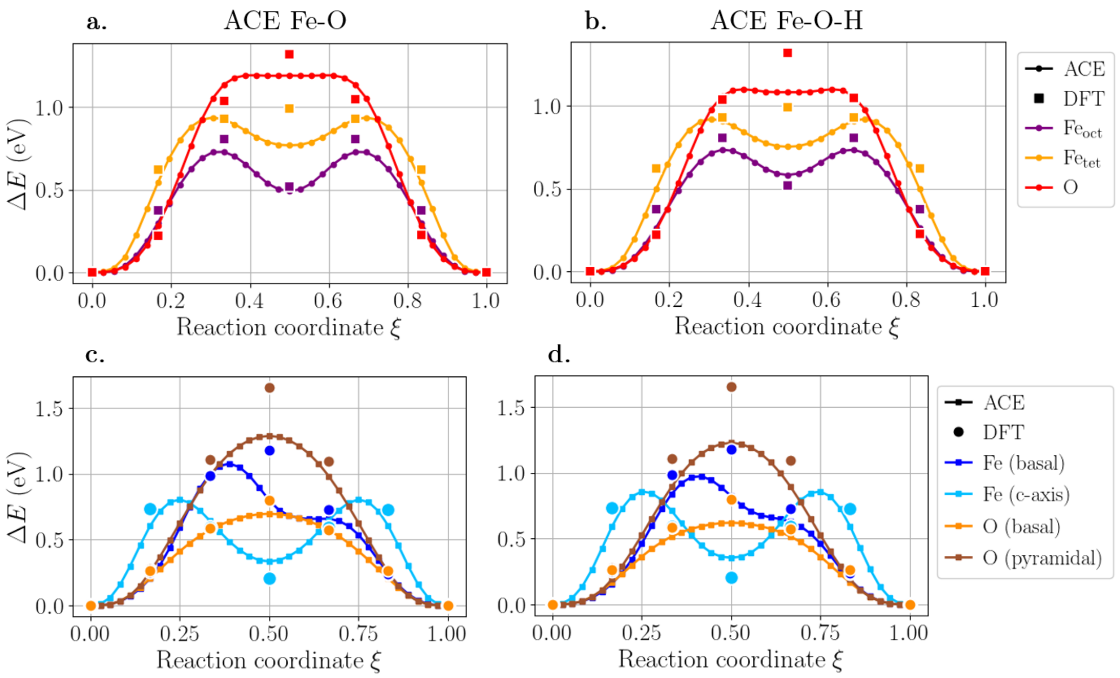

Supplementary Figure 6: Diffusion of Fe and O vacancies in (**a-b**) Fe_3O_4, and (**a-b**) Fe_2O_3 obtained with the binary Fe-O (left column) and ternary Fe-O-H (right column) ACE potentials, and also compared to DFT calculations (symbols).

We also present in Supplementary Fig. 7 point defects formation energies as a function of the oxygen chemical potential for BCC Fe (Fe vacancy and O interstitial), and the two Fe_3O_4 and Fe_2O_3 iron oxides (Fe and O vacancies and interstitials), as predicted by the two binary Fe-O and ternary Fe-O-H ACE potentials, showing the good agreement found between the two models.

For BCC Fe (Supplementary Fig. 7 **a**), we use a $5 \times 5 \times 5$ supercell in the three [100], [010] and [001] directions of the conventional BCC unit-cell. For Fe_3O_4 (Supplementary Fig. 7 **b**), a $2 \times 2 \times 2$ supercell was used, along the three [100], [010] and [001] directions of the conventional cubic spinel unit-cell. And for Fe_2O_3 (Supplementary Fig. 7 **c**), a $2 \times 1 \times 1$ supercell was used, along the three $[1\bar{1}00]$, $[11\bar{2}0]$ and [0001] axis of the conventional orthorhombic cell. Lowest energy configurations for Fe and O interstitials in both iron oxides, *i.e.* Fe_3O_4 and Fe_2O_3, are the identical for both ACE potentials, and correspond to the ones reported in our previous work on the binary Fe-O system [1].

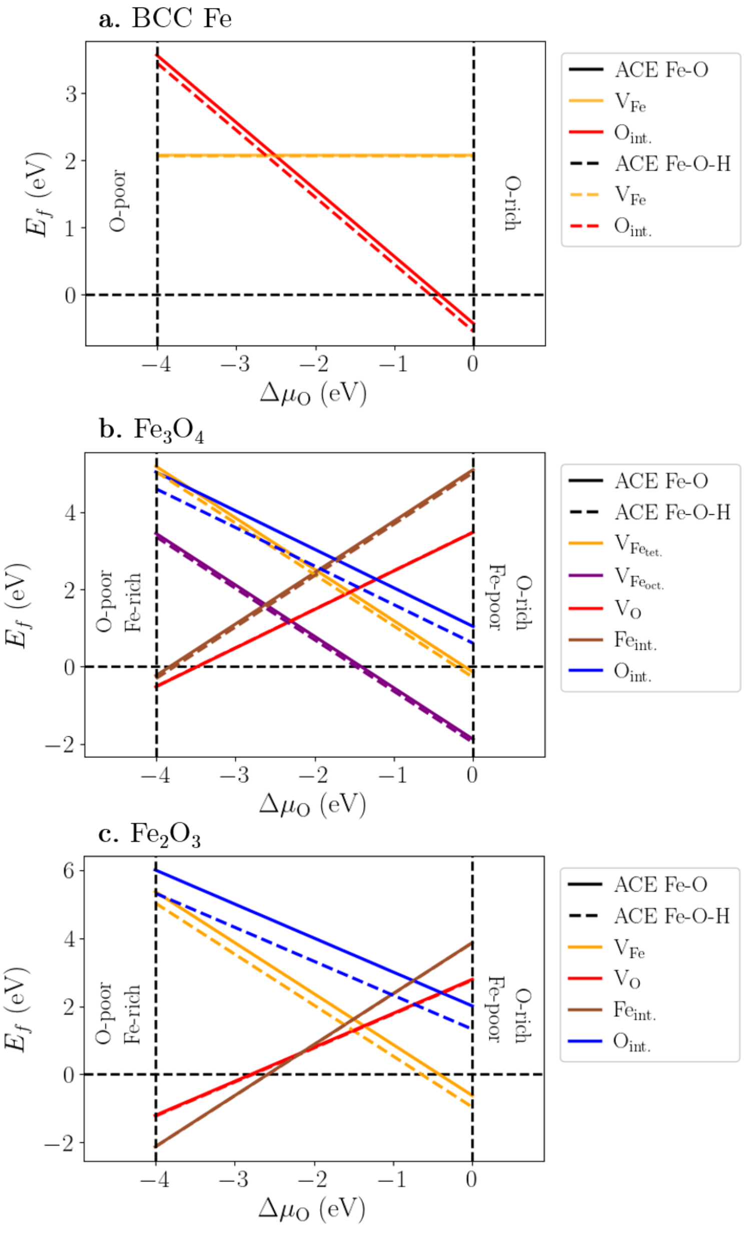

Supplementary Figure 7: Point defects formation energies E_f as a function of the oxygen chemical potential $\Delta\mu_O$, referenced to molecular O_2, in (**a**) BCC Fe, (**b**) Fe_3O_4, and (**c**) Fe_2O_3, as predicted by the binary Fe-O (full lines) and ternary Fe-O-H (dashed lines) ACE potentials. V_{Fe}: Fe vacancy (also with subscripts "tet." and "oct." in Fe_3O_4, referring to tetrahedral and octahedral Fe) ; V_O: O vacancy : $Fe_{int.}$: Fe interstitial ; $O_{int.}$: O interstitial.

Supplementary Note S3: Clean grain boundary energies in BCC Fe

We present in Supplementary Fig. 8 the energy of clean grain boundaries in BCC Fe, focusing on tilt boundaries with rotation axis of [001], [011] and [111], comparing results of the binary Fe-O ACE potential [1], the present ternary Fe-O-H ACE potential, and DFT calculations taken from Ref. [12]. Both grains on the two sides of the grain boundaries are at least 20 Å-thick, and every structure is first annealed at 600 K for 1 ps before being quenched down to 0 K for energy evaluation. Predictions of the ternary ACE potential compare very well with DFT calculations, for all three rotation axis considered. We still note that if the energy spectra in Supplementary Fig. 8 might appear to show "rough" parts, it might be caused by the structures considered not being fully relaxed to the lowest energy minimum. Also, only grain boundaries defined by $\Sigma \leq 60$ are considered.

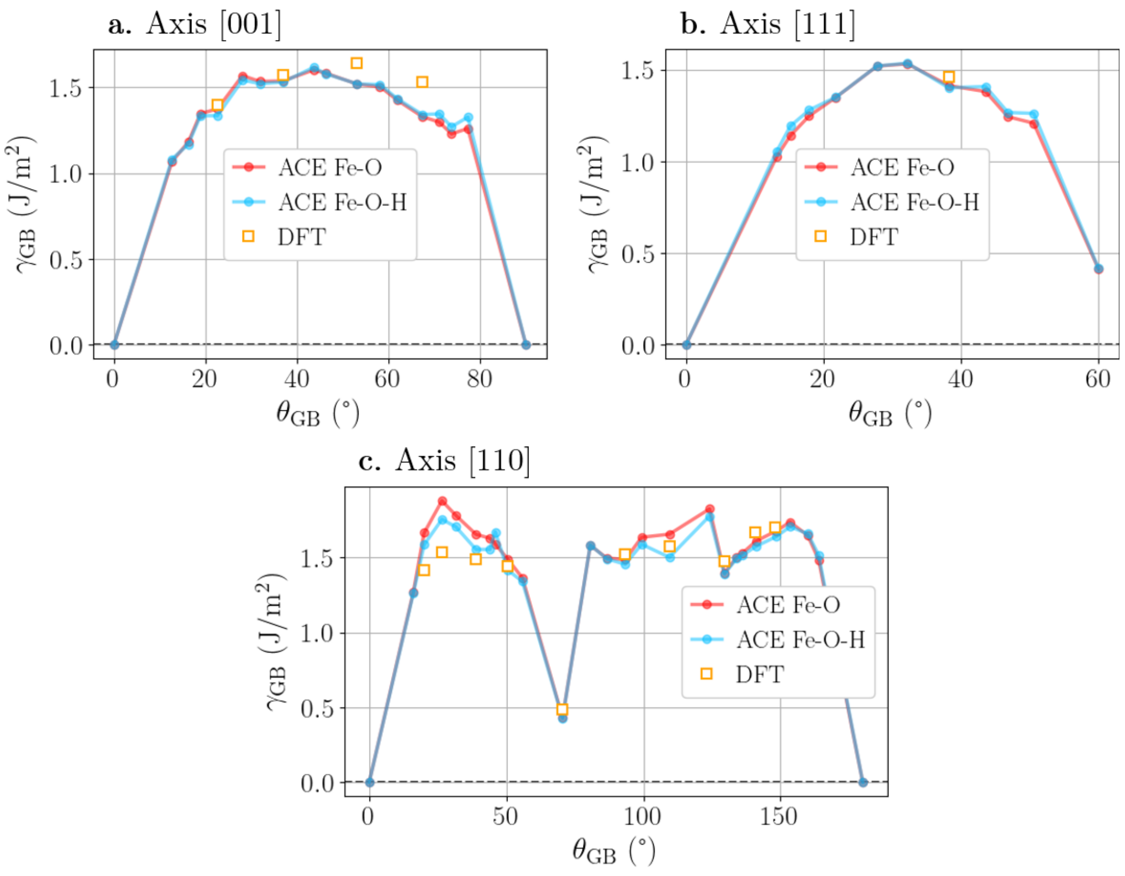

Supplementary Figure 8: Symmetric tilt grain boundary energies γ_{GB} in BCC Fe as a function of their misorientation angle θ_{GB} for a (**a**) [001], (**b**) [111], and (**c**) [110] rotation axis. Predictions of both the binary Fe-O (red) and ternary Fe-O-H (light blue) ACE potentials are compared, with DFT references taken from Refs. [12, 13].

Supplementary Note S4: Impact of magnetic order on selected properties

Magnetic order and disorder for various structures

We present in Supplementary Fig. 9 the formation enthalpy of various prototype materials, namely BCC Fe, Fe_2O_3 and FeO(OH), as a function of atomic volume for different magnetic states. For each one of these materials, we considered its ground state magnetic order (FM for BCC Fe, AF for FeO(OH) and Fe_2O_3), the FM order, and 50 different random distributions of spins up and down on the iron sublattice.

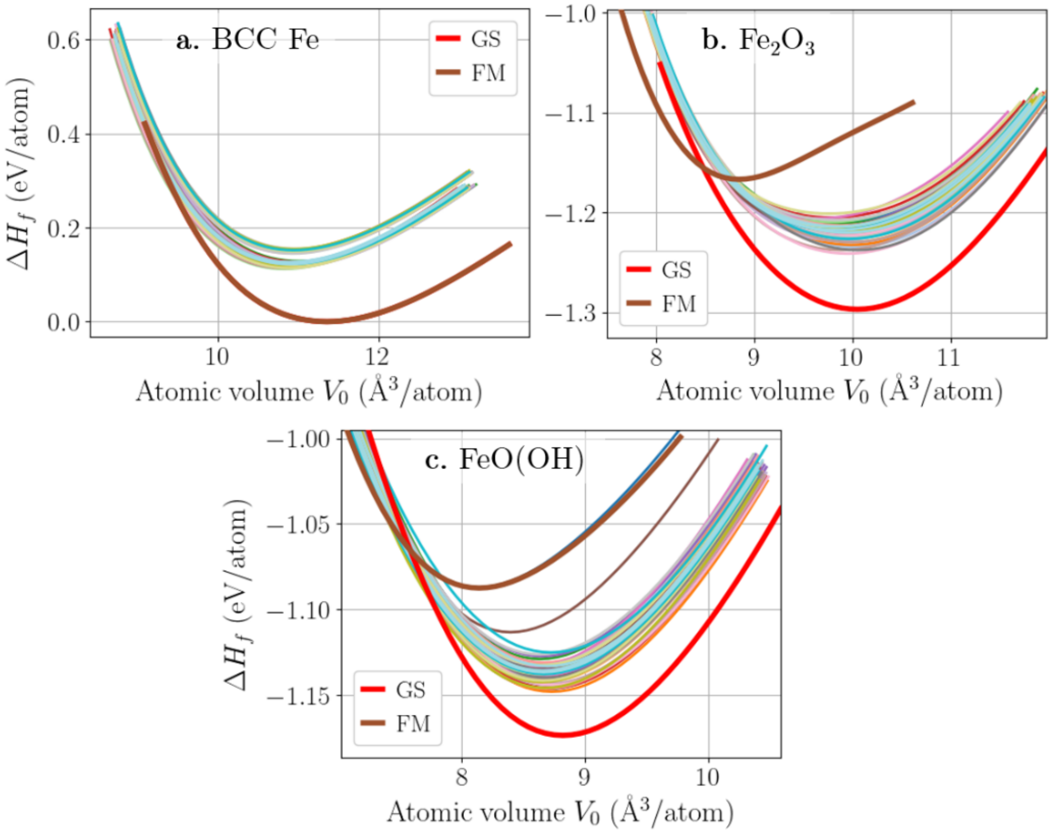

Supplementary Figure 9: Formation enthalpy as a function of atomic volume for **(a)** BCC Fe, **(b)** Fe_2O_3 and **(c)** FeO(OH), considering various magnetic orderings: the ground state (GS) of each structure (in red), namely ferromagnetic (FM) for BCC Fe, and antiferromagnetic (AF) for Fe_2O_3 and FeO(OH), FM (in brown), and 50 different random magnetic configurations (in other colors).

The ACE potential captures the energy variations as a function of both volume and magnetic order, across a wide variety of prototypes. For each of the three structures considered in Supplementary Fig. 9, we observe that accounting for the FM order instead of the magnetic ground state of the structure, would yield a different equilibrium volume

and formation enthalpy of the material.

Impact of the treatment of magnetism on diffusion

We now study the impact of magnetic order on the diffusion of Fe interstitials (crucial for the phase transformation between various iron oxides) in Fe_3O_4, using different "treatments" of magnetic degrees of freedom within the framework of the ACE potential. In particular, we considered three different initializations of the magnetic order: the ground state FeM, FM and random arrangements of spins on the Fe sublattice. Additionally, we also investigated the impact of switching on the on-the-fly spin equilibration taking the FeM order as a benchmark. For each of these four setups, 5 different Fe_3O_4 supercells containing randomly distributed Fe interstitial atoms were generated. All simulations ran for 2 ns with a timestep of 1 fs at temperatures of 600, 800, 1000, 1200 and 1400 K. We present in Supplementary Fig. 10 the measured mean-squared displacement (MSD) of Fe atoms as a function of simulation time along the various trajectories, to the slope of which the effective diffusivity of Fe interstitials is directly proportional.

We observe that allowing for spin equilibration and starting from the ground state FeM order of Fe_3O_4 (see Supplementary Fig. 10 a) yields a much more consistent picture of diffusion of Fe interstitials in the matrix, with a small scatter between different configurations at all considered temperatures. When the magnetic order is instead kept fixed (comparing to Supplementary Fig. 10 b), the MSD reaches similar values, but a significant scatter in the data is observed among different starting configurations. Now fixing the magnetic structure to FM (Supplementary Fig. 10 c) or various random arrangements of spins (Supplementary Fig. 10 d) yields different a diffusivity for Fe interstitials.

Most importantly, we note that, except for simulations at the FeM ground state of Fe_3O_4, simulation cells started to melt below the highest considered temperature of 1400 K, in particular between 1000 and 1200 K for fixed FM structures, and between 1200 and 1400 K for fixed random spin arrangements, which is why MSD data at these temperatures are not presented in Supplementary Fig. 10. This premature melting under incorrect magnetic order is itself evidence that an accurate treatment of magnetism is not merely beneficial, but necessary for thermodynamically stable simulations in the Fe-O-H ternary system.

All simulations were performed using a $3 \times 3 \times 3$ supercell of the conventional cell of Fe_3O_4 with axis aligned along [100], [010] and [001], containing a total of 1512 atoms. For

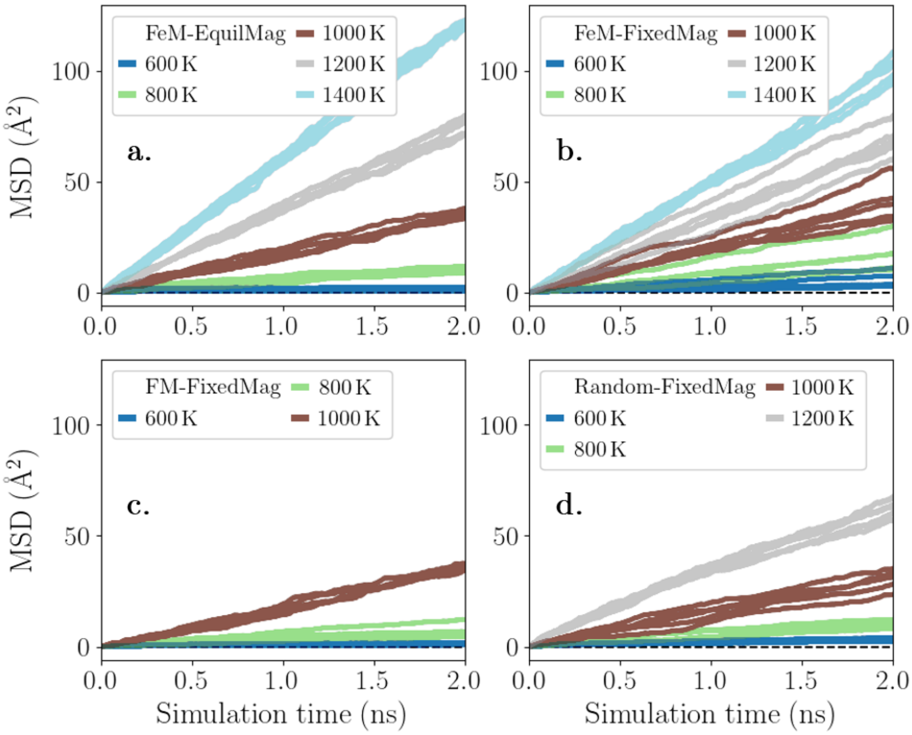

Supplementary Figure 10: Mean-squared displacement (MSD) of Fe atoms as a function of simulation time, computed along a MD trajectory of a Fe_3O_4 supercell containing Fe interstitial atoms. The impact of the treatment of magnetism is shown here considering the FeM magnetic ground state of Fe_3O_4 **(a)** with on-the-fly equilibration of the spins of Fe atoms ("FeM-EquilMag") and **(b)** without (*i.e.* fixed magnetic species, "FeM-FixedMag"), **(c)** fixed magnetic order to FM ("FM-FixedMag"), and **(d)** random arrangements of spins on the Fe sublattice, which is kept fixed ("Random-FixedMag"). For each condition (*i.e.* magnetism and temperature), five different initial structures were used.

each of the simulation conditions (*i.e.* treatment of magnetism), 5 different configurations seeded with randomly arranged Fe interstitial atoms were generated. The concentration of these atoms accounts for 1 % of the total number of Fe atoms in the bulk supercell, *i.e.* 6 interstitial Fe atoms.

Supplementary Note S5: Properties of liquids and near-melting conditions

Density of iron-oxygen liquids

We present in Supplementary Fig. 11 the density of liquid Fe-O systems at various oxygen concentrations at 2000 K predicted by the ACE potential, and compared to both experimental data and results from various reaxFF potentials. Data from experiments and reaxFF potentials are taken from [14].

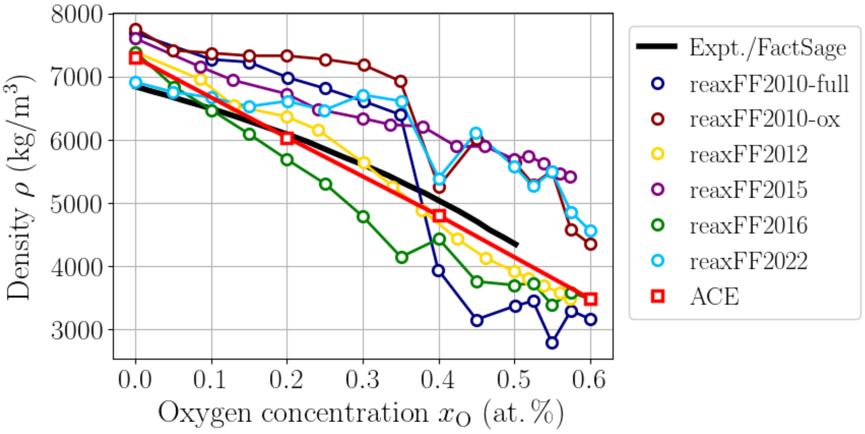

Supplementary Figure 11: Density ρ (in kg/m^3) of liquid iron and iron oxides at 2000 K as a function of the oxygen concentration x_O (in at. %) as predicted by the Fe-O-H ACE potential presented in this work (red squares). Results are compared to experimental data and calculated using FactSage (in black) and to predictions of various reaxFF potentials (other symbols), taken from [14].

The agreement of the predictions of the ACE potential with experimental data, as opposed to the scatter shown among the reaxFF parameterizations, shows the applicability of the ACE potential to high temperature liquids, as well as the transferability of the model. Along the MD trajectories, the spins of the Fe atoms are allowed to swap between magnetic species handled by the ACE potential, in order to equilibrate magnetic degrees of freedom as well. We note that across the oxygen concentration range covered, all liquid structures strongly prefer disordered magnetic configurations with random distribution of spin up and spin down species across the system. We also performed these simulations with a fixed ferromagnetic order, and present in Supplementary Fig. 12 the evolution of the energy of the system and the radial distribution functions, comparing with the trajectory along which the magnetic order was equilibrated. For this example, the same starting structure for liquid iron-oxygen

with 60 % of oxygen ($Fe_{0.4}O$) was picked.

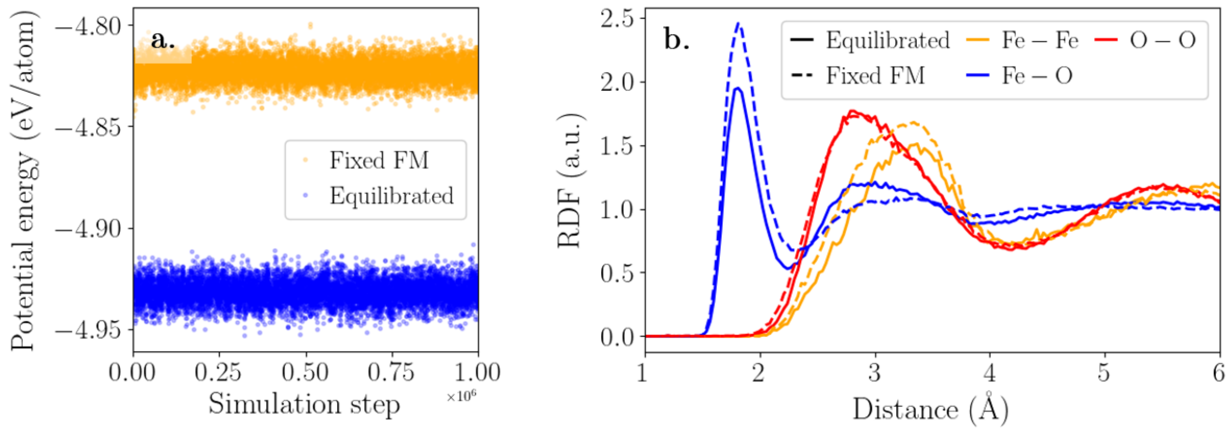

Supplementary Figure 12: Impact of switching on magnetic equilibration along a MD trajectory of a $Fe_{0.4}O$ liquid (*i.e.* with 60 % of oxygen) at zero pressure and 2000 K: **(a)** potential energy per atom as a function of simulation steps (with a timestep of 1 fs), and **(b)** partial radial distribution functions (RDF) averaged over the 2 ns of the simulation as a function of distance, comparing a trajectory with spin equilibration (full lines) and a magnetic order fixed to FM (dashed lines).

As presented in Supplementary Fig. 12 a, allowing for on-the-fly equilibration of the iron spins allows to reach more energetically favorable structures, with an average energy decrease of 110 meV/atom with respect to a fixed FM magnetic order. The structure of the two $Fe_{0.4}O$ liquids, as described by the partial radial distribution functions presented in Supplementary Fig. 12 b, are similar, but a slight shift in the peak positions and magnitude of the Fe-Fe and Fe-O distributions is observed, showing the difference in the liquid structures predicted along the two MD trajectories.

Dehydroxylation of goethite, FeO(OH)

We present in Supplementary Fig. 13 the decomposition process of goethite, FeO(OH), near the transition temperature. At medium temperatures, FeO(OH) decomposes into hematite (Fe_2O_3) and water vapor (H_2O), a process termed dehydroxylation. We studied this complex process, involving both chemical and structural transformations and phase changes, along a MD trajectory at 1000 K in order to further demonstrate the transferability of the proposed ACE potential. Along the simulation, we monitored the extrapolation grade, which stayed within the interpolation regime.

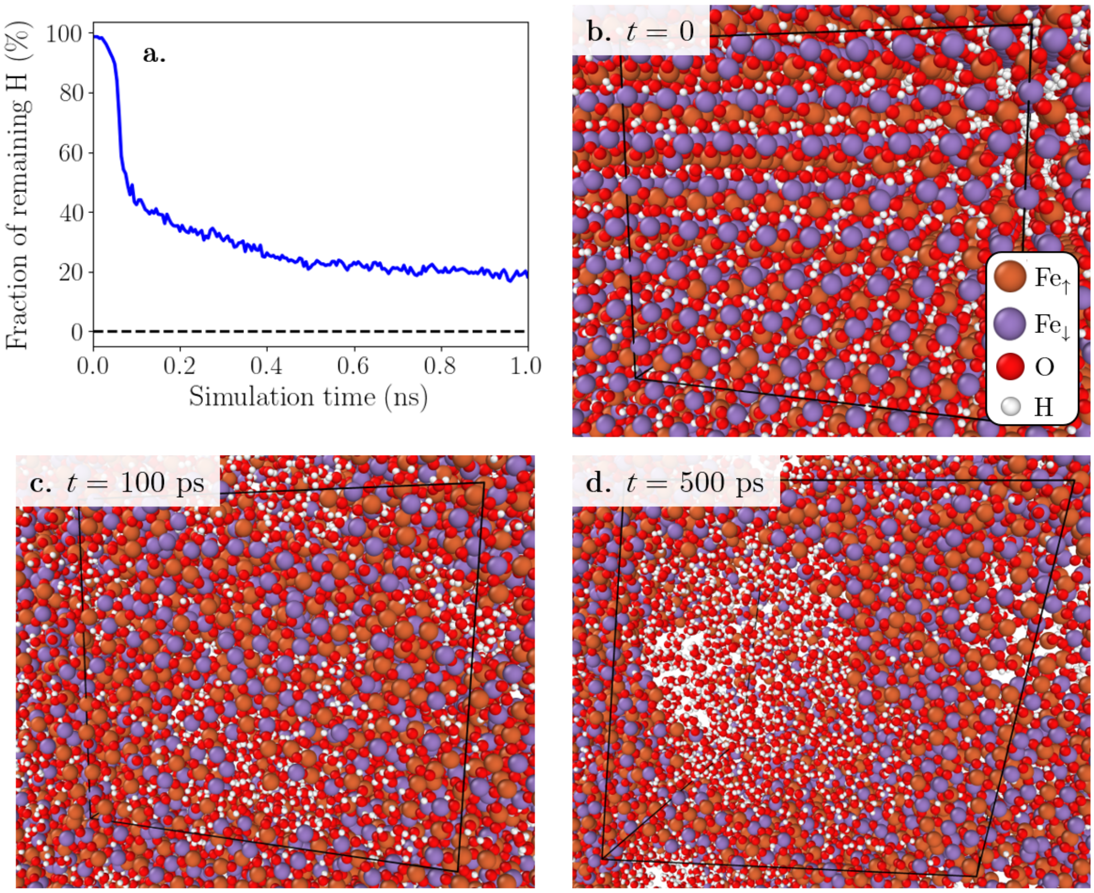

Supplementary Figure 13: MD trajectory of the decomposition/dehydroxylation of goethite FeO(OH): **(a)** fraction of H atoms remaining in the solid phase as a function of simulation time, **(b-d)** snapshots of the simulation at different times. Fe atoms are colored in orange and purple, depending on the sign of their magnetic moment, O atoms are in red, and H atoms in white. For visualization purposes, only a 40 Å-thick slice of the whole system is presented.

We also quantify the dehydroxylation process by evaluating the fraction of H atoms remaining in the solid phase, presented in Supplementary Fig. 13 a, which corresponds to the formation of H_2O molecules leaving the solid phase while taking O atoms away. We also present snapshots along the MD trajectory in Supplementary Fig. 13 b-d, showing the formation of H_2O molecules as the initial FeO(OH) decomposes into a water-rich gas phase and an iron/oxygen-rich solid phase.

Supplementary Note S6: Interaction energies between dislocation cores and interstitial O and H atoms in BCC Fe

DFT simulation setup

For computing interaction energies between between dislocations and interstitial O and H atoms, we use a different setup for the dislocations than the DFT-compatible quadrupole, namely a periodic array of dislocations [15]. In this setup, the simulation cell is periodic in the directions of the dislocation line and glide direction (*e.g.* [111] and $[\bar{1}12]$ for the $\frac{1}{2}[111]$ screw dislocation on Fig. 5 **a** of the main text), but not in the direction normal to plane (*e.g.* $[1\bar{1}0]$ on Fig. 5 **a** of the main text). This method has been applied for computing interaction energies between a $\frac{1}{2}[111]$ screw dislocation and interstitial O atoms in Ref. [16] and H atoms in Ref. [17], for instance. The DFT setup is presented in Supplementary Fig. 14.

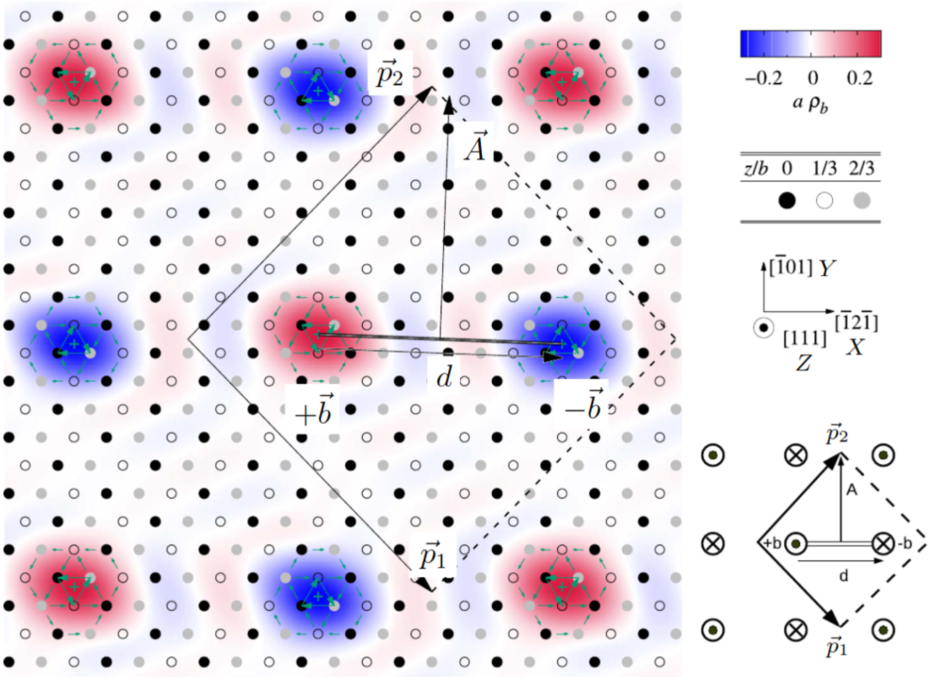

Supplementary Figure 14: Simulation setup for modeling a $\frac{1}{2}[111]$ screw dislocation in BCC Fe. The cell contains a periodic array of screw dislocations with opposite Burgers vectors. Atoms are colored differently depending on their height along the [111] direction. Arrows between atoms, representing the differential displacement between them along [111] caused by the dislocation core, indicated by green crosses. The superimposed color map corresponds to the Nye tensor, a measure of the dislocation density (reproduced from [18]).

Interstitial atoms, here O or H, are placed in the vicinity of both dislocation cores, having

opposite Burgers vectors, and the interaction energy $E_{\text{int.}}^{\text{dislo-O/H}}$ is then obtained as:

$$E_{\text{int.}}^{\text{dislo-O/H}} = \frac{1}{2}\left(E^{\text{dislo+O/H}} - E^{\text{dislo}}\right) - \left(E^{\text{bulk+O/H}} - E^{\text{bulk}}\right), \tag{1}$$

where $E^{\text{dislo+O/H}}$ is the total energy of the simulation cell containing two symmetrically identical O or H atoms close to the dislocation cores, E^{dislo} is the energy of the same simulation cell without O or H atom. $E^{\text{bulk+O/H}}$ and E^{bulk} are the total energies of bulk BCC Fe with and without an interstitial O or H atom in its most favorable configuration.

Reconstruction of the $1/2\langle 111\rangle$ *screw dislocation core*

Dislocation-solute interaction energies presented in the main text were in the dilute case, *i.e.* with one interstitial atom along a dislocation line at least 7 Å-long. In this section, we instead study the interaction energies for a fully decorated dislocation line, *i.e.* with one or more solute elements per unit length of dislocation line, as presented in Supplementary Fig. 15. In this regime, DFT calculations have demonstrated that solute elements like H, O, C or N can drive a reconstruction of the $1/2\langle 111\rangle$ screw dislocation core, from its ground-state easy configuration (see Supplementary Fig. 15 **a**) to the hard core configuration (see Supplementary Fig. 15 **b**) [16, 17].

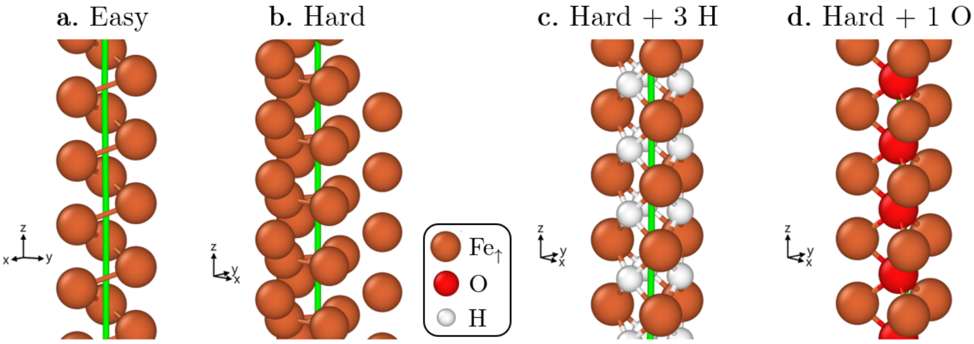

Supplementary Figure 15: Different configurations of the $1/2\langle 111\rangle$ screw dislocation core: (**a**) easy and (**b**) hard core configurations, without solute atoms. Reconstructed core from the easy to the hard configuration in the presence of (**c**) 3 H and (**d**) 1 O atom per unit length of dislocation line. Fe atoms are in orange, O in red, and H in white, respectively. The green lines represent the dislocation lines. The OVITO package [19] was used for visualization.

For these calculations, the DFT-compatible geometry presented in Supplementary Fig. 14 is used, where solute atoms are placed in the vicinity of both dislocation cores having

opposite Burgers vectors. We start with both easy and hard configurations of the core, and systematically find that the presence of 3 H atoms or 1 O atom per unit length of dislocation line drives the reconstruction of the easy core to the hard configuration, as presented in Supplementary Fig. 15 **c** and **d**, respectively.

Supplementary Note S7: Surface-specific phase diagram of BCC Fe with adsorbed H and O atoms

We present in Supplementary Fig. 16 the {210}-specific surface phase diagram of BCC Fe with H atoms, as discussed in the main text in the context of the MD simulation of the dissociation and subsequent penetration of H atoms through GBs. We also present surface-specific phase diagrams of various BCC Fe facet orientations with absorbed H atoms in in Supplementary Fig. 17, and O atoms in in Supplementary Fig. 18.

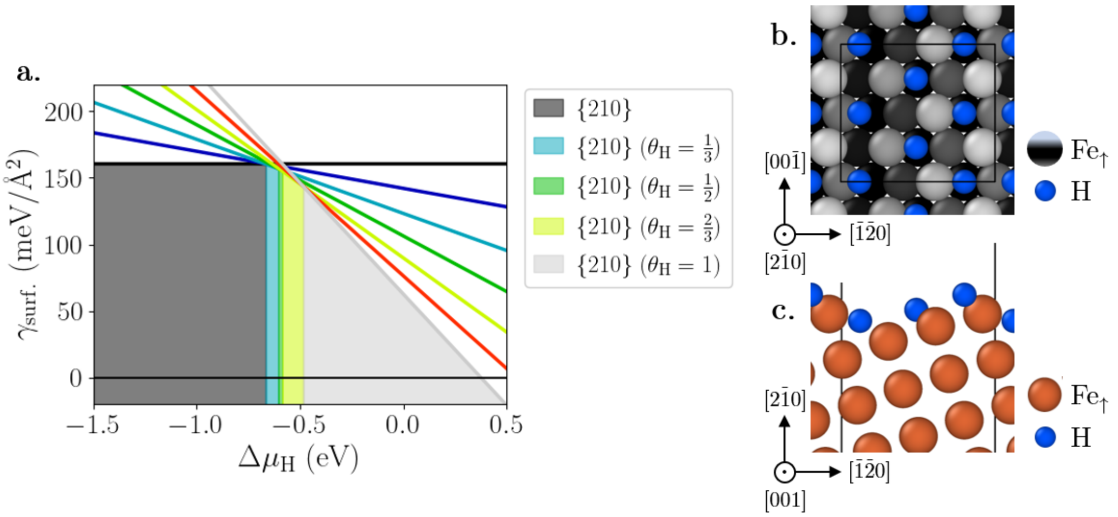

Supplementary Figure 16: (**a**) Phase diagram of the {210} surface of BCC Fe covered by hydrogen at different surface coverages θ_{H}. The vertical green line indicates the approximate H chemical potential corresponding to the H_2 pressure used in the MD simulation presented in Fig. 8 of the main text. Top (**b**) and side (**c**) views of the fully covered ($\theta_{\mathrm{H}} = 1$) {210} surface. On the top view, Fe atoms are colored in black to white as they get closer to the surface. On the side view, Fe atoms are in orange. H atoms are in blue.

As discussed in the main text, we also compute the coverage-dependent adsorption energies of both O and H adsorbated on various surfaces of BCC Fe, presented in Supplementary Fig. 19. Interestingly, we observe repulsive interactions between adsorbed H atoms for different facets, leading to an increase of the adsorption energy per H atom $E^{\mathrm{H}}_{\mathrm{ads.}}$ as a function of surface coverage θ_{H}. For the {100}, {110}, {111} and {320} surfaces, such interactions are attractive, with a varying equilibrium coverage at which the minimum adsorption energy per surface H atom is found. A similar phenomena is also observed considering surface O adsorbates (see Supplementary Fig. 19 **b**).

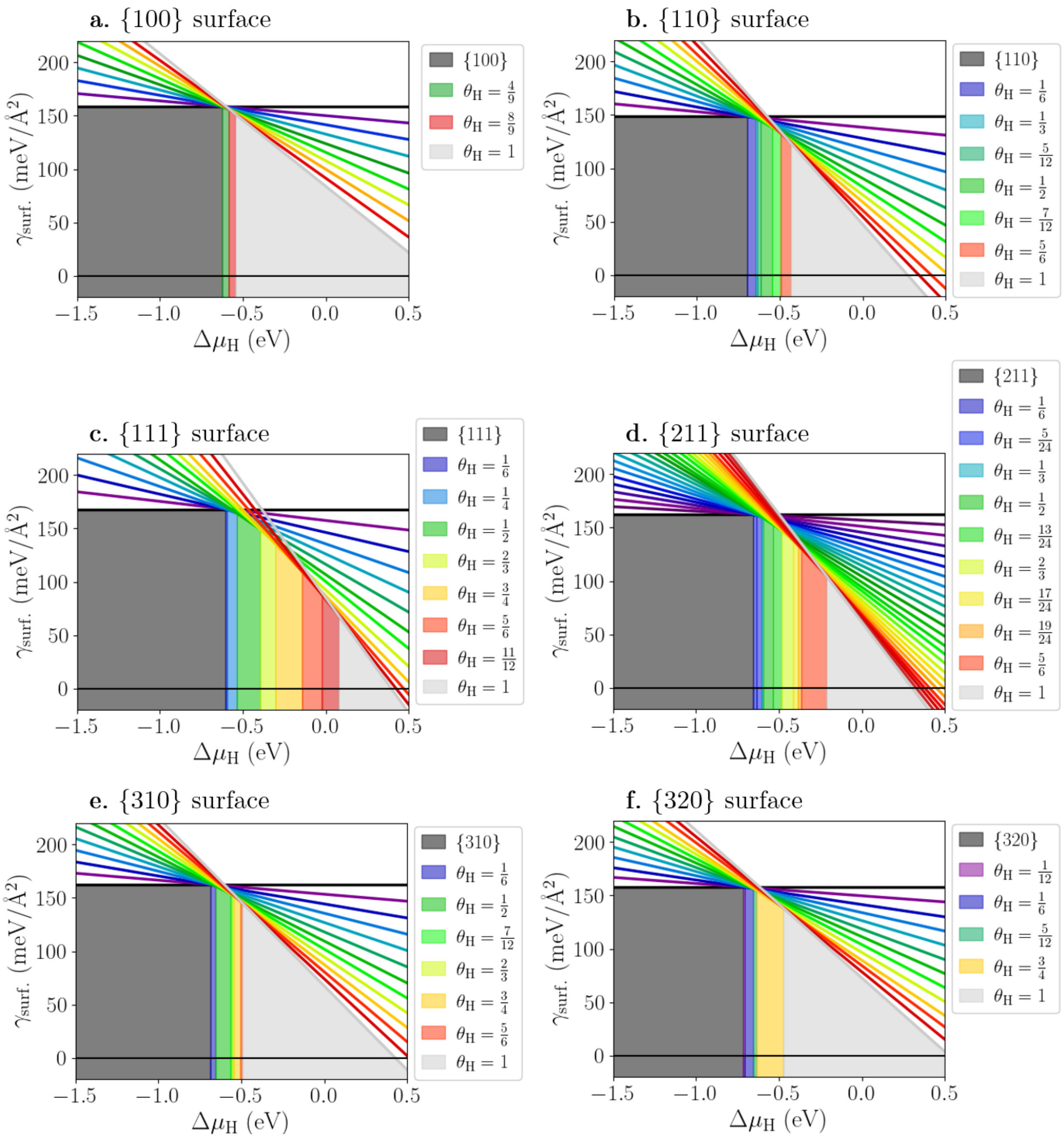

Supplementary Figure 17: Phase diagram of different surfaces of BCC Fe covered by hydrogen at different surface coverages θ_H: (**a**) {100}, (**b**) {110}, (**c**) {111}, (**d**) {211}, (**e**) {310}, (**f**) {320}. Only labels for the stable surface coverages are present on each phase diagram.

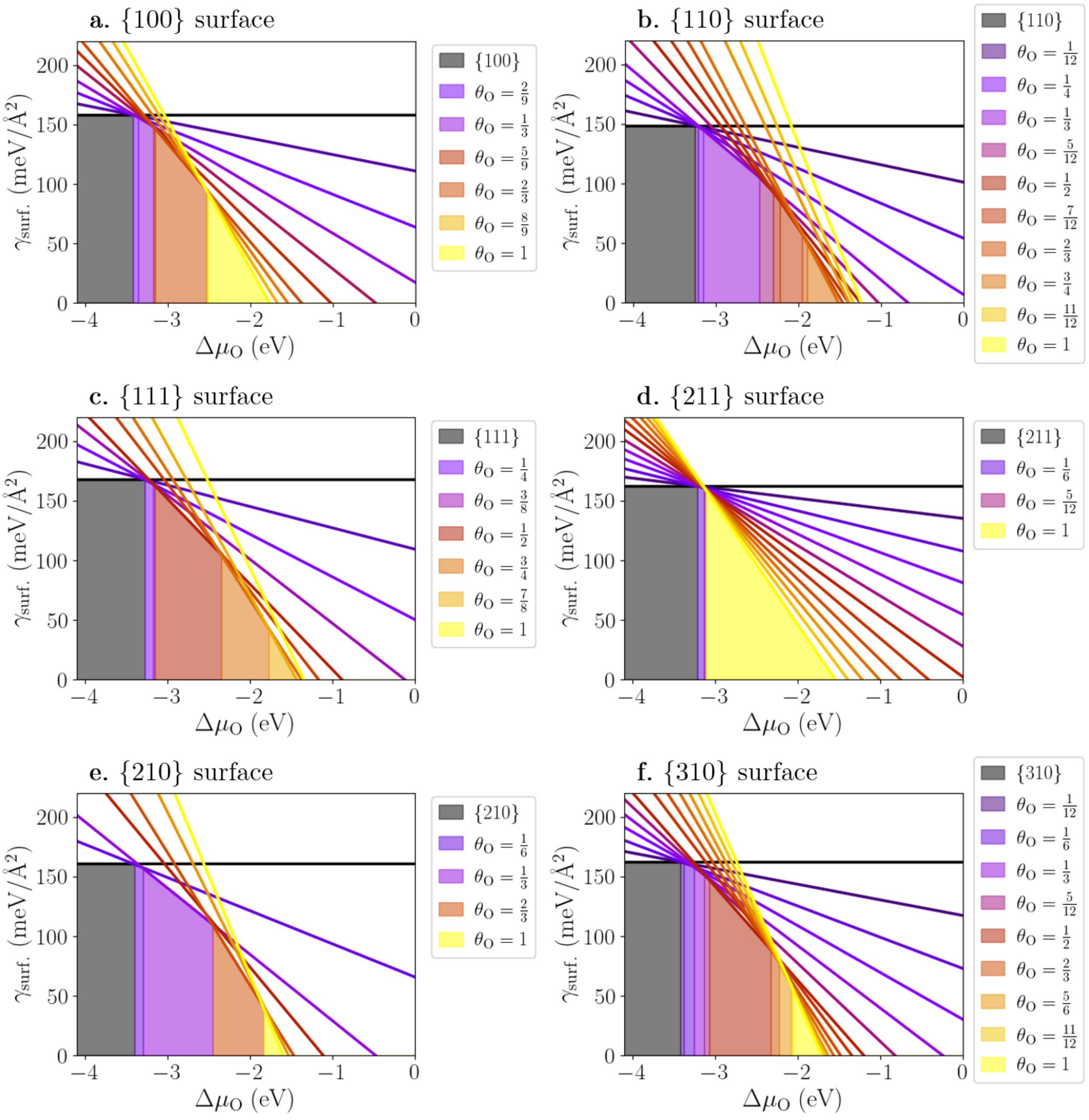

Supplementary Figure 18: Phase diagram of different surfaces of BCC Fe covered by oxygen at different surface coverages θ_{O}: (**a**) {100}, (**b**) {110}, (**c**) {111}, (**d**) {211}, (**e**) {210}, (**f**) {310}. Only labels for the stable surface coverages are present on each phase diagram.

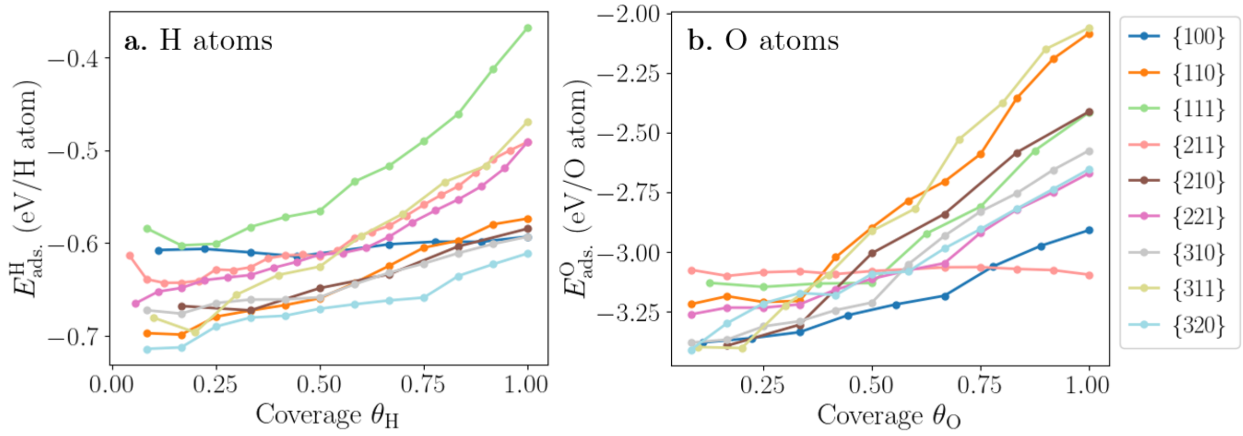

Supplementary Figure 19: Adsorption energy $E_{ads.}$ of (**a**) H, and (**b**) O atoms on various surfaces of BCC Fe as a function of the surface coverages in H/O atoms $\theta_{H/O}$.

Supplementary Note S8: Simulation setups and details

We detail in this section simulation setups (*i.e.* geometry, parameters, number of atoms) and methods used for both validation and applications of the ternary Fe-O-H ACE potential presented in the main text. All DFT calculations were performed using the VASP code [20], while atomistic simulations with the developed ACE potential were performed using both the LAMMPS code [21] and the Atomic Simulation Environment (ASE) [22], supporting ACE potentials as implemented in Refs. [23–25].

Section III A 2

In Fig. 1, point defects (vacancies and H interstitials) were evaluated in the following setup: for vacancies in FeH polymorphs (Fig. 1 **a**), we used a $2 \times 2 \times 2$ supercell of the conventional cubic cell of the FCC phase, and a $3 \times 3 \times 1$ supercell of the primitive hexagonal cell for the HCP and DHCP phases ; for vacancies in FeO(OH) (Fig. 1 **b**), a $3 \times 2 \times 1$ supercell of the conventional orthogonal cell was used ; for H interstitial atoms (Fig. 1 **c**), we used a $4 \times 4 \times 4$ supercell of the conventional cubic cell of for BCC Fe, a $6 \times 6 \times 4$ supercell of the body-centered tetragonal cell for FCC Fe (AFDL), a $5 \times 3 \times 3$ supercell of the orthogonal cell for HCP Fe (AF), a $2 \times 2 \times 2$ supercell of the conventional cubic cell of Fe_3O_4, and a $2 \times 1 \times 1$ supercell of the rhombohedral cell of Fe_2O_3.

Section III B 1

In Fig. 2 **a**, interstitial H binding energies with both vacancies ($\mathrm{V_{Fe}} - \mathrm{H_{int.}}$) and a vacancy-interstitial O atom complex ($V_{\mathrm{Fe}} - \mathrm{O_{int.}} - \mathrm{H_{int.}}$) in BCC Fe were computed in a $3 \times 3 \times 3$ supercell of the conventional cubic cell of BCC Fe. In Fig. 2 **b**, the coupled diffusion of a vacancy-interstitial H atom complex ($\mathrm{V_{Fe}} - \mathrm{H_{int.}}$) was studied in a $4 \times 4 \times 4$ supercell of the conventional cubic cell of BCC Fe. The migration barrier was obtained using the nudged elastic band (NEB) method as implemented in the LAMMPS package [21], with a spring constant of $5\,\mathrm{eV/Å^2}$ between adjacent images along the path. These images are then relaxed until forces fall below a threshold of $5\,\mathrm{meV/Å}$ along all three Cartesian directions.

Section III B 2

In Fig. 3 **a**, for the diffusion of interstitial H atoms in the three polymorphs of pure Fe (*i.e.* BCC, FCC and HCP), we used a $4 \times 4 \times 4$ supercell of the conventional cubic

cell of the BCC phase (FM), a $6 \times 6 \times 4$ supercell of the body-centered tetragonal cell for FCC Fe (AFDL), a $3 \times 2 \times 2$ supercell of the orthogonal cell for HCP Fe (AF). As for H diffusion in Fe_3O_4 (Fig. 3 **b**) a $2{\times}2{\times}2$ supercell of the conventional cubic cell is used.

Section III D

Regarding MD simulations presented in Section III D, we used the geometries described here.

For the H_2-driven reduction of the Fe_2O_3 basal (0001) surface (Fig. 7 of the main text), the simulation cell contains a 28 Å-thick symmetric slab exposing two identical O-rich facets, made of 4032 Fe and 6552 O atoms, exposed to a gas of 1680 H_2 molecules, accounting for a hydrogen pressure $P_{H_2} = 1$ kbar at the 1000 K temperature of the MD simulation. Along the trajectory, a barostat at zero pressure is applied to the in-plane directions of the simulation cell, and Fe atoms are allowed to swap their spins between spin up and down species, as described in our previous work on the ACE potential for the Fe-O system [1].

For the MD simulation of BCC Fe surface permeation and diffusion, presented in Fig. 8 of the main text, the simulation cell contains a total of 13200 and 11952 Fe atoms for the two parallel ($\|$) and perpendicular ($\perp$) setups, respectively. Slab models are 31 Å-thick, and expose two identical $\{210\}$ surfaces, containing one and two $\Sigma5[001](2\bar{1}0)$ grain boundaries, for the two $\|$ and $\perp$ setups used in the simulations, respectively. A hydrogen pressure of approximately $P_{H_2} = 1$ kbar at the 1000 K temperature of the MD simulation is then put in contact with the two geometries filling vacuum regions by 2816 and 2704 H_2 molecules, for the $\|$ and $\perp$ setups, respectively. A barostat is then applied to the in-plane directions of the simulation cell along the MD trajectory.

Finally, for the MD simulation of a BCC Fe surface in contact with H_2O vapor, presented in Fig. 9 of the main text, the cell contains a 30 Å-thick symmetric slab of 9828 Fe atoms exposing two identical $\{320\}$ surfaces to a vapor containing 1428 H_2O molecules, accounting for a water density $\rho_{H_2O} = 0.40\,\mathrm{g/cm^3}$. Along the MD trajectory at 1000 K, a barostat is applied to the two in-plane directions of the simulation cell.

Supplementary Note S9: Structure of the DFT training set

We detail in this section the content of the DFT dataset used for training the ACE potential. We first present in Supplementary Fig. 20 the extent of the entire DFT training set for the Fe-O-H ACE potential presented in this work, showing the coverage in terms of formation enthalpy as a function of nearest neighbor distance. Structures pertaining to different chemical subsets of the system (*e.g.* Fe-only, binary Fe-O or ternary Fe-O-H) are plotted in different colors.

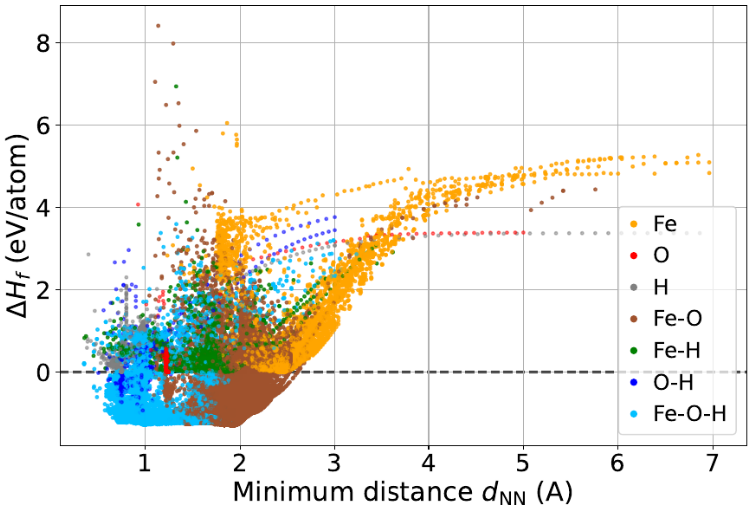

Supplementary Figure 20: Formation enthalpy ΔH_f (in eV/atom) as a function of nearest neighbor distance $d_{\rm NN}$ for structures spanning the entire DFT dataset used for training the ACE Fe-O-H potential. Structures pertaining to each subset of the Fe-O-H ternary system (*e.g.* pure Fe, Fe-O, Fe-O-H) are indicated by different colors.

Across the entire dataset, pure iron (Fe, in orange on Supplementary Fig. 20), oxygen (O, in red on Supplementary Fig. 20) and hydrogen (H, in grey on Supplementary Fig. 20) are sampled by 7549, 1032 and 1022 structures, respectively. For the binary sub-systems, iron-oxygen (Fe-O, in brown on Supplementary Fig. 20) is sampled by 18689 structures, iron-hydrogen (Fe-H, in green on Supplementary Fig. 20) by 7967 structures, and oxygen-hydrogen (O-H, in blue on Supplementary Fig. 20) by 1810 structures. For the subset containing all three chemical species (Fe-O-H, in light blue on Supplementary Fig. 20), 24500 structures are used to sample it. All structures in subsets containing iron species are then included twice in the final dataset, both their original spin configuration as well as their spin-inverse (*i.e.* all spins on all Fe atoms are swapped, with then corresponds to

the same energy). This last step accounts for the spin-inversion symmetry that the ACE potential needs to respect. More details are also given in our previous work supporting the model for the binary Fe-O model. In total, the dataset contains about 122000 structures. All structures lying below a maximum of 10 eV/atom from the convex Hull and with force components below 100 eV/Å are used for training.

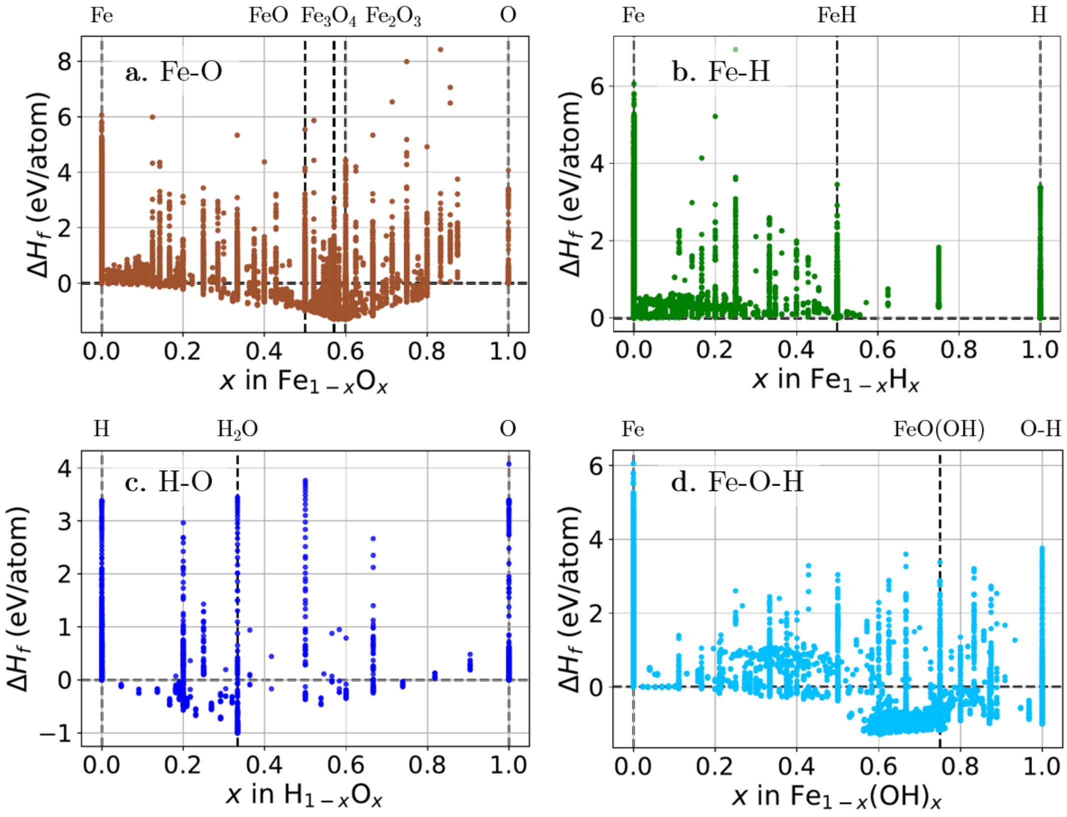

Supplementary Figure 21: Formation enthalpy ΔH_f (in eV/atom) as a function of composition for entire training set across various separate sub-systems: **(a)** Fe-O, **(b)** Fe-H, **(c)** H-O and **(d)** Fe-O-H. Each subset contains only structures with a combination of the specified elements, *i.e.* all Fe-O-H structures contain at least one atom of the three Fe, O and H species. Black vertical dashed lines indicate the location of various prototype structures discussed in the main text, also representing the most "relevant" materials covered by the Fe-O-H system.

We also present in Supplementary Fig. 21 the formation enthalpy of all structures contained in the training set for various subsets of the ternary Fe-O-H system, illustrating how broadly the training data samples the compositional space of the Fe-O-H ternary system. Main representative prototypes on which more emphasis was put (*i.e.* experimentally known

and stable materials) are located by black vertical dashed lines.

We also show in Supplementary Table III details of the different types of structures included in the training set, for each of the chemical subsets presented in Supplementary Fig. 20. We classify these structures into different categories: bulk prototypes (where the more emphasis was put) with perturbations (with rattling of atoms and random deformations), explicit defects (*e.g.* vacancies, interstitials, surfaces, dislocations, grain boundaries, interfaces…), ASSYST-like structures (*i.e.* structures generated from randSpg [26], pyxtal [27] and/or buildcell [28]), molecular/cluster configurations, random collinear magnetic configurations (for Fe-containing structures only), and active learning loops.

Supplementary Table III: Number and types of structures over the entire training set for the Fe-O-H ACE potential presented in this work. For Fe-containing subsets (*i.e.* Fe, Fe-O, Fe-H and Fe-O-H), the "total" number of configurations are included twice in the final dataset, one for each spin-reversed symmetry-equivalent, to account for spin-inversion symmetry.

Subsets	Fe	O	H	Fe-O	Fe-H	O-H	Fe-O-H
Bulk + perturbed:	4918	0	0	5507	1029	0	732
Explicit defects:	1274	0	0	5209	4345	0	19982
ASSYST-like:	392	0	0	5967	1008	0	2169
Molecules/clusters:	345	1028	966	66	11	1768	239
Magnetic states:	527	0	0	403	0	0	0
Active learning:	0	4	56	0	66	42	1053
Miscellaneous:	93	0	0	1537	1508	0	325
**Total:**	**7549**	**1032**	**1022**	**18689**	**7967**	**1810**	**24500**

As can be noted, most of the structures are obtained following a similar approach to the ASSYST method [29, 30], which has been shown to effectively sample diverse atomic environments, such as the core of complex defects (*e.g.* dislocations, grain boundaries) or highly deformed structures (*e.g.* occurring along phase transformations) without explicitly including these structures into the training set. We note here that within the "ASSYST-like" structures, magnetic degrees of freedom are sampled as well, since all structures generated using these approaches are considered with various initializations of the magnetic moments on Fe atoms. As for "Active learning", these also sampled a diverse part of the configura-

tional space, since they were both generated along MD trajectories where the extrapolation grade was initially high, as well as for generating structures from a methodology close to ASSYST.

In the Fe-O-H subset (Supplementary Fig. 21 **d**), most structures correspond to Fe-O structures (*i.e.* iron oxides) with different content of hydrogen, also sampling chemical reactions at the surface, and also iron reactions with water. In addition, it contains a number of ASSYST-like structures, as detailed above and in Supplementary Tab. III. As for O, H and O-H subsets, most of the structures correspond to mixtures of gases (with more emphasis put on H_2O, O_2 and H_2), random molecular clusters, and random arrangements of the two chemical species. The Fe, O and Fe-O subsets are mostly sampled using the training set for our previous model for the Fe-O binary system [1].

Supplementary Note S10: Model performance and limitations

Computational cost

We now compare the performance in terms of computational cost of our developed ACE potential to other available models covering parts of the ternary Fe-O-H system, presented in Tab. IV. For this purpose, we performed MD simulations under NVT conditions at 300 K. For Fe, we used a 432-atom supercell of BCC Fe, for Fe-H a 512-atom supercell of FCC FeH, for Fe-O a 448-atom supercell of Fe_3O_4, and for Fe-O-H a 480-atom supercell of FeO(OH). All simulations were ran for 100 ps, with a timestep of 1 fs, on a single CPU.

Supplementary Table IV: Computational cost (in 10^3 atoms.step/second) compared between different interatomic potentials across various sub-systems of the ternary Fe-O-H. Details about the simulation details are given in the text.

Model	Fe	Fe-H	Fe-O	Fe-O-H
EAM2006 [31]	466.9	/	/	/
EAM2021 [32]	521.6	228.1	/	/
MEAM2007 [33]	102.3	57.3	/	/
ADP2022 [34]	138.9	62.3	/	/
ABOP2019 [35]	36.8	/	41.3	/
reaxFF2010 [36]	6.8	6.9	4.9	3.8
**ACE (this work)**	**4.7**	**5.2**	**4.1**	**4.6**

We also note that upon on-the-fly equilibration of the spins of the Fe sublattice, a decrease of the performance proportional to the attempt frequency of the Monte Carlo (MC) moves is observed. For instance, the parameters for this procedure in presented simulations is 100 MC swap attempts each 10 steps of the underlying MD, in order to ensure the system is in its magnetic ground state at the given temperature of the MD. Thus, a slow down of $100/10 = 10$ is observed as compared to the same simulation with a fixed magnetic order.

Limitations and dangerous cases

We stress that even if the present ACE potential for the Fe-O-H system is aimed at describing a wide variety of situations hosted within the ternary system, some situations might still be

problematic. In particular, potential shortcomings are expected under "extreme" conditions of temperature and/or pressure, especially when fast-moving H atoms are contained in the simulation. For such cases, potential users are advised to carefully monitor stability of the simulations, shortest distances between atom pairs, and exploration of spurious atomic environments along the trajectory.

Supplementary references

[1] B. Bienvenu, M. Todorova, J. Neugebauer, D. Raabe, M. Mrovec, Y. Lysogorskiy, and R. Drautz, npj Comput. Mater. **11**, 1 (2025).

[2] Z. S. Basinski, W. Hume-Rothery, and A. L. Sutton, Proc. R. Soc. London **229**, 459 (1955).

[3] M. Rinaldi, M. Mrovec, A. Bochkarev, Y. Lysogorskiy, and R. Drautz, npj Comput. Mater. **10**, 1 (2024).

[4] J. J. Adams, D. S. Agosta, R. G. Leisure, and H. Ledbetter, J. Applied Phys. **100**, 113530 (2006).

[5] L. De Schepper, D. Segers, L. Dorikens-Vanpraet, M. Dorikens, G. Knuyt, L. M. Stals, and P. Moser, Phys. Rev. B **27**, 5257 (1983).

[6] H. J. Wollenberger, Physical Metall. **2** (1996).

[7] J. Zhang, Phys. Rev. Lett. **84**, 507 (2000).

[8] D. R. Stull and H. Prophet, *JANAF Thermochemical Tables, Second Edition*, NBS Technical Note NSRDS-NBS 37 (National Bureau of Standards, 1971).

[9] C. Haavik, S. Stølen, H. Fjellvåg, M. Hanfland, and D. Häusermann, American Mineralogist **85**, 514 (2000).

[10] L. W. Finger and R. M. Hazen, J. App. Phys. **51**, 5362 (1980).

[11] B. Bienvenu, L. Dezerald, D. Rodney, and E. Clouet, Acta Mater. **236**, 118098 (2022).

[12] F.-S. Meng, J.-P. Du, S. Shinzato, H. Mori, P. Yu, K. Matsubara, N. Ishikawa, and S. Ogata, Phys. Rev. Mater. **5**, 113606 (2021).

[13] J. Wang, G. K. H. Madsen, and R. Drautz, Modelling Simul. Mater. Sci. Eng. **26**, 025008 (2018).

[14] L. C. Thijs, E. M. Kritikos, A. Giusti, M.-A. van Ende, A. C. T. van Duin, and X. Mi, J. Phys. Chem. A **127**, 10339 (2023).

[15] D. Bacon, Y. Osetsky, and D. Rodney, in *Chapter 88 Dislocation–Obstacle Interactions at the Atomic Level*, Dislocations in Solids, Vol. 15 (Elsevier, 2009) pp. 1–90.

[16] B. Lüthi, L. Ventelon, D. Rodney, and F. Willaime, Comput. Mater. Sci. **148**, 21 (2018).

[17] P. P. P. O. Borges, E. Clouet, and L. Ventelon, Acta Mater. **234**, 118048 (2022).

[18] E. Clouet, B. Bienvenu, L. Dezerald, and D. Rodney, Comptes Rendus. Physique **22**, 83 (2021).

[19] A. Stukowski, Modelling and Simulation in Materials Science and Engineering **18**, 015012 (2009).

[20] G. Kresse and J. Furthmüller, Comput. Mater. Sci. **6**, 15 (1996).

[21] A. P. Thompson, H. M. Aktulga, R. Berger, D. S. Bolintineanu, W. M. Brown, P. S. Crozier, P. J. in 't Veld, A. Kohlmeyer, S. G. Moore, T. D. Nguyen, R. Shan, M. J. Stevens, J. Tranchida, C. Trott, and S. J. Plimpton, Comput. Phys. Com. **271**, 108171 (2022).

[22] A. Hjorth Larsen, J. Jørgen Mortensen, J. Blomqvist, I. E. Castelli, R. Christensen, M. Dułak, J. Friis, M. N. Groves, B. Hammer, C. Hargus, E. D. Hermes, P. C. Jennings, P. Bjerre Jensen, J. Kermode, J. R. Kitchin, E. Leonhard Kolsbjerg, J. Kubal, K. Kaasbjerg, S. Lysgaard, J. Bergmann Maronsson, T. Maxson, T. Olsen, L. Pastewka, A. Peterson, C. Rostgaard, J. Schiøtz, O. Schütt, M. Strange, K. S. Thygesen, T. Vegge, L. Vilhelmsen, M. Walter, Z. Zeng, and K. W. Jacobsen, J. Phys.: Condensed Matter **29**, 273002 (2017).

[23] R. Drautz, Phys. Rev. B **99**, 10.1103/PhysRevB.99.014104 (2019).

[24] Y. Lysogorskiy, C. v. d. Oord, A. Bochkarev, S. Menon, M. Rinaldi, T. Hammerschmidt, M. Mrovec, A. Thompson, G. Csányi, C. Ortner, and R. Drautz, npj Comput. Mater. **7**, 1 (2021).

[25] A. Bochkarev, Y. Lysogorskiy, S. Menon, M. Qamar, M. Mrovec, and R. Drautz, Phys. Rev. Mater. **6**, 013804 (2022).

[26] P. Avery and E. Zurek, Comput. Phys. Communications **213**, 208 (2017).

[27] S. Fredericks, K. Parrish, D. Sayre, and Q. Zhu, Computer Phys. Comm. **261**, 107810 (2021).

[28] C. J. Pickard and R. J. Needs, J. Phys. Cond. Mat. **23**, 053201 (2011).

[29] M. Poul, L. Huber, E. Bitzek, and J. Neugebauer, Phys. Rev. B **107**, 104103 (2023).

[30] M. Poul, L. Huber, and J. Neugebauer, npj Comput. Mater. **11**, 174 (2025).

[31] H. Chamati, N. Papanicolaou, Y. Mishin, and D. Papaconstantopoulos, Surf. Sci. **600**, 1793 (2006).

[32] M. Wen, Comput. Mater. Sci. **197**, 110640 (2021).

[33] B.-J. Lee and J.-W. Jang, Acta Mater. **55**, 6779 (2007).

[34] S. Starikov, D. Smirnova, T. Pradhan, I. Gordeev, R. Drautz, and M. Mrovec, Phys. Rev. Mater. **6**, 043604 (2022).

[35] J. Byggmästar, M. Nagel, K. Albe, K. O. E. Henriksson, and K. Nordlund, J. Phys. Cond. Mat. **31**, 215401 (2019).

[36] M. Aryanpour, A. C. T. van Duin, and J. D. Kubicki, J. Phys. Chem. A **114**, 6298 (2010).
